# Identification and Characterization of Metastasis‐Initiating Cells in ESCC in a Multi‐Timepoint Pulmonary Metastasis Mouse Model

**DOI:** 10.1002/advs.202401590

**Published:** 2024-06-12

**Authors:** Ching Ngar Wong, Yu Zhang, Beibei Ru, Songna Wang, Hongyu Zhou, Jiarun Lin, Yingchen Lyu, Yanru Qin, Peng Jiang, Victor Ho‐Fun Lee, Xin‐Yuan Guan

**Affiliations:** ^1^ Department of Clinical Oncology Centre for Cancer Medicine Li Ka Shing Faculty of Medicine The University of Hong Kong Hong Kong SAR 999077 China; ^2^ Department of Pediatric Oncology Sun Yat‐sen University Cancer Center Guangzhou 510060 China; ^3^ State Key Laboratory of Oncology in South China Sun Yat‐sen University Cancer Center Guangzhou 510060 China; ^4^ Cancer Data Science Lab Center for Cancer Research National Cancer Institute National Institutes of Health Bethesda MD 20814 USA; ^5^ Department of Clinical Oncology the First Affiliated Hospital Zhengzhou University Zhengzhou 450052 China; ^6^ Department of Clinical Oncology The University of Hong Kong ‐ Shenzhen Hospital Shenzhen 518053 China; ^7^ Shenzhen Key Laboratory for cancer metastasis and personalized therapy The University of Hong Kong‐Shenzhen Hospital Shenzhen 518053 China; ^8^ Advanced Energy Science and Technology Guangdong Laboratory Huizhou 516029 China; ^9^ MOE Key Laboratory of Tumor Molecular Biology Jinan University Guangzhou 510610 China

**Keywords:** early cancer metastasis, early metastasis microenvironment, esophageal squamous cell carcinoma, metastasis biomarkers, metastasis‐initiating cells, multiplex staining, single‐cell transcriptome sequencing

## Abstract

Metastasis is the biggest obstacle to esophageal squamous cell carcinoma (ESCC) treatment. Single‐cell RNA sequencing analyses are applied to investigate lung metastatic ESCC cells isolated from pulmonary metastasis mouse model at multiple timepoints to characterize early metastatic microenvironment. A small population of parental KYSE30 cell line (Cluster S) resembling metastasis‐initiating cells (MICs) is identified because they survive and colonize at lung metastatic sites. Differential expression profile comparisons between Cluster S and other subpopulations identified a panel of 7 metastasis‐initiating signature genes (MIS), including CD44 and TACSTD2, to represent MICs in ESCC. Functional studies demonstrated MICs (CD44^high^) exhibited significantly enhanced cell survival (resistances to oxidative stress and apoptosis), migration, invasion, stemness, and in vivo lung metastasis capabilities, while bioinformatics analyses revealed enhanced organ development, stress responses, and neuron development, potentially remodel early metastasis microenvironment. Meanwhile, early metastasizing cells demonstrate quasi‐epithelial‐mesenchymal phenotype to support both invasion and anchorage. Multiplex immunohistochemistry (mIHC) staining of 4 MISs (CD44, S100A14, RHOD, and TACSTD2) in ESCC clinical samples demonstrated differential MIS expression scores (dMISs) predict lymph node metastasis, overall survival, and risk of carcinothrombosis.

## Graphical abstract

CD44, TACSTD2, S100A14, and RHOD act as signatures to represent cancer‐spreading‐initiating cells in esophageal squamous cell carcinoma. These cells acquire both mobile and stational properties that flavor spreading and anchorage. High signature score predicts poor patient clinical outcomes, including worsen overall survival, increased risk of cancer‐spreading in lymph node, and increased risk of blood vessel blockage by spreading cancer cells.

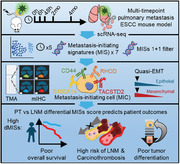

## 1 Introduction

Esophageal squamous cell carcinoma (ESCC) is the major subtype of esophageal carcinoma with high mortality rate due to frequent relapse and metastasis.^[^ ^1^ ^]^ Five‐year survival rate of metastatic patients is less than 5%.^[^ ^2^ ^]^ Lung is the most common distant metastatic organ (11.4%) in ESCC, with different characteristics from other metastatic sites.^[^ ^2^ ^]^ Cancer heterogeneity has been evinced in a variety of solid tumors including ESCC.^[^ ^3^ ^]^ and acts as a cancer hallmark indicating the importance of genome instability in tumor progression.^[^ ^4^ ^]^ Intertumor heterogeneity has been widely studied in different ESCC patients to identify common genetic alterations that promote tumor progression,^[^ ^4^, ^5^, ^6^, ^7^ ^]^ while intratumor heterogeneity in identifying subpopulations within ESCC tumors is an emerging field of research in cancer metastasis. It is believed that cancer metastasis originates from a small tumor subpopulation with unique characteristics, which refers to intratumor heterogeneity in gene expression profiles,^[^ ^8^, ^9^ ^]^ and involves multiple simultaneous or sequential steps, including dissemination, colonization, and dormancy,^[^ ^10^, ^11^ ^]^ required for migration and invasion into and out of circulations and extracellular matrix (ECM), anchorage, proliferation of tumor cells, resistance to various stresses, as well as to establish a metastasis favorable niche.^[^ ^12^, ^13^, ^14^ ^]^ This small subpopulation of tumor cells is called metastasis‐initiating cells (MICs) because of its ability to form distant organ metastases.

Therefore, identification and characterization of MICs are crucial for understanding mechanism of tumor metastasis as well as for early detection and intervention to reduce metastasis‐related mortality. However, identification of MICs in ESCC is inadequate, especially at early metastatic stage.^[^ ^15^, ^16^ ^]^ Recently, we established a mouse lung metastasis model and isolated metastatic tumor cells from lungs at multiple timepoints for single‐cell RNA‐sequencing (scRNA‐seq) analysis to identify potential MICs with enhanced survival and metastatic properties, together with their representative metastasis‐initiating signatures (MIS) as biomarkers. Then, in silico, in vitro and in vivo experiments were performed using flow sorted MIS‐enriched subpopulations to investigate cell survival (resistances to oxidative stress and apoptosis), cell migration, invasion, stemness, and in vivo lung metastasis capabilities. Finally, clinical relevance of MISs was tested as predictive biomarkers for patient outcomes by multiplex immunohistochemistry (mIHC) staining and statistical analyses based on co‐expression patterns of MISs in cell lines, metastatic mice lungs, and ESCC tissue microarray (TMA).

## 2 Results

### 2.1 Establishment of a Pulmonary Metastatic Mouse Model with Multi‐Time Tracking

**Figure 1:**
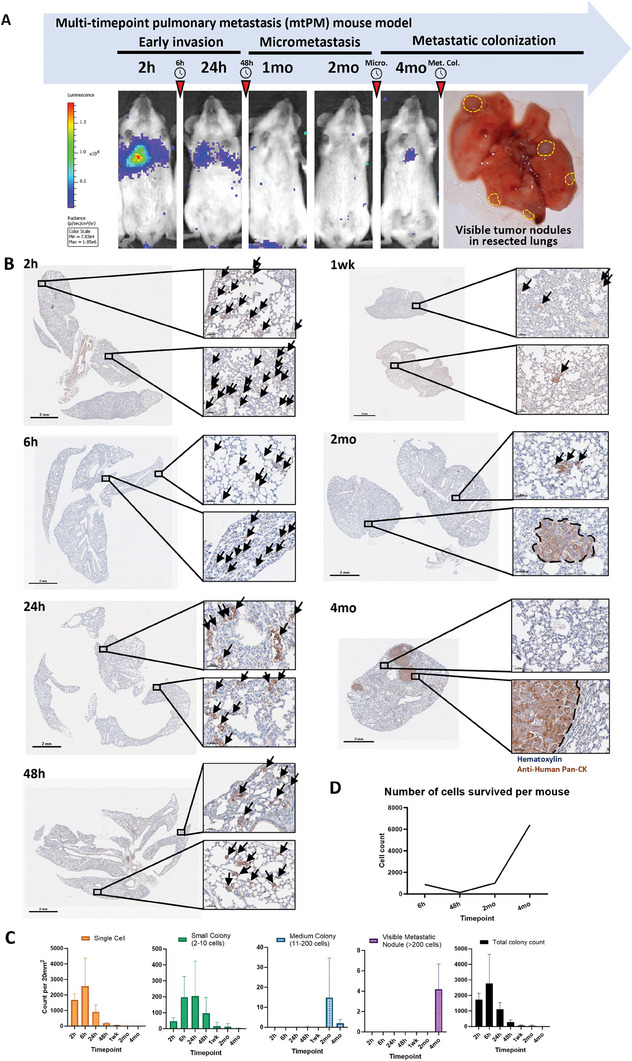
Multi‐timepoint pulmonary metastasis (mtPM) ESCC mouse model establishment. A) Representative in vivo bioluminescence images of mtPM mouse model (at 2 h, 24 h, 1 mo, 2 mo, and 4 mo) and resected mice lung. Mice were intravenously inoculated with KYSE30‐Luc‐GFP ESCC cells. Bioluminescence scaled from 7.83e4 to 1.85e6 radiance (p/sec/cm^2^/sr). Yellow dashed circles indicated metastasized tumor nodules in lungs. Red arrows indicated collection timepoints for single‐cell RNA sequencing. B) Representative immunohistochemistry (IHC) staining of metastasized tumor cells in formalin‐fixed and paraffin‐embedded (FFPE) mice lungs post‐intravenous inoculation at 2 h (*n* = 3), 6h (*n* = 3), 24 h (*n* = 4), 48 h (*n* = 4), 1wk (*n* = 3), 2 mo (*n* = 4), and 4 mo (*n* = 4). Human pan‐Cytokeratin stained brown. Black arrows and dashed circles indicated tumor cells. Left panel: Whole lung (scale bar = 2 mm). Right small panels: Zoomed‐in sections (scale bar = 50 µm). C) Bar charts showing number of colony counts per 20 mm square of mice lung tissue section. Four colony sizes were determined at cross‐sectional plane. Bar charts from left to right represents single cell, small colony (2‐10 cells), medium colony (11‐200 cells), visible metastatic nodule (>200 cells), and total colony count. D) Line graph showing number of tumor cells survived per mice. Flow cytometry analysis of GFP positive living tumor cells from each freshly dissociated mice lungs collected from four timepoints, including 6 h, 48 h, 2 mo, and 4 mo.

To investigate early‐stage metastatic microenvironment of ESCC in vivo, we utilized luciferase and green fluorescent protein labeled ESCC cell line (KYSE30‐Luc‐GFP) to establish a pulmonary metastatic mouse model with multi‐time tracking. KYSE30‐Luc‐GFP cells (1.2 × 10^6^) were injected into NOD SCID mice via tail vein, and bioluminescence signals in lungs were monitored longitudinally at multiple indicated timepoints (2 h, 6 h, 24 h, 48 h, 1 week, 2 months and 4 months). This mouse model exhibited dynamic in vivo bioluminescence signal expression along the timeline, with a gradual decrease in signal observed after inoculation, eventually becoming undetectable at 1 month and reappeared at 4 months with visible metastasis in resected lungs (**Figure** **1**A).

### 2.2 Detection of Dynamic Viable Metastatic Tumor Cells in Mouse Model

To quantify surviving tumor cells in lungs injected intravenously, viable tumor cells in lungs at different timepoints were detected by immunohistochemistry (IHC) staining and counted by flow cytometry. IHC staining of human pan‐Cytokeratin (pan‐CK) was performed to count survival metastatic colonies in mouse lungs at seven timepoints included 2 h, 6 h, 24 h, 48 h, 1 week, 2 months, and 4 months (Figure 1B). Based on tumor cell numbers, metastatic colonies were divided into single cell, small (2‐10 cells), medium (11‐200 cells), and large (>200 cells) metastatic colonies (Figure 1C). The results showed tumor cells initially seeded in the lungs as single cells or small colonies within 6 h, and then number of colonies decreased and most survivors proliferated as small colonies at 24 h. Most of the seeded single cells and small colonies died within 48 h and maintained low proliferative capacity through week 1. Tumor colonies were further reduced at 2 months, mostly medium colonies, and visible metastatic nodules were observed at 4 months. We therefore hypothesize that there are two major crises for early metastatic survival: overcoming stresses and establishing metastasis favoring niches. First, most circulating tumor cells (CTCs) die due to their inability to withstand various stresses on their way to distant organs, such as loss of anchorage in circulation (i.e., anoikis), oxidative stresses, and immune attack.^[^ ^9^, ^17^, ^18^ ^]^ Second, tumor cells need to establish a special early metastasis microenvironment to help them implant into lung tissue and form metastatic colonies, while preventing immune attacks and obtaining nutrients.

Next, flow cytometry was performed to calculate number of GFP‐positive living tumor cells in the lungs of each mouse at 6 h, 48 h, 2 months, and 4 months (Figure 1D). Results showed tumor cells bottomed out at 48 h, gradually rebounded at 2 months, and rocketed exponentially at 4 months, confirming the existence of a temporary survival crisis on day 2, and increased cell proliferation in the micrometastasis state at 2 months. It is worth noting that multi‐timepoint IHC series provides a more accurate picture of the dynamic survival tumor cells in the lungs compared with flow cytometry analysis. In summary, multi‐timepoint IHC series and flow cytometry analysis found that most CTCs survived for a few days in the lungs, and only a small proportion of CTCs that overcome the early metastatic survival crisis are able to maintain latency and eventually form metastatic nodules.

### 2.3 Identification of Metastasis‐Initiating Cells (MICs)

**Figure 2:**
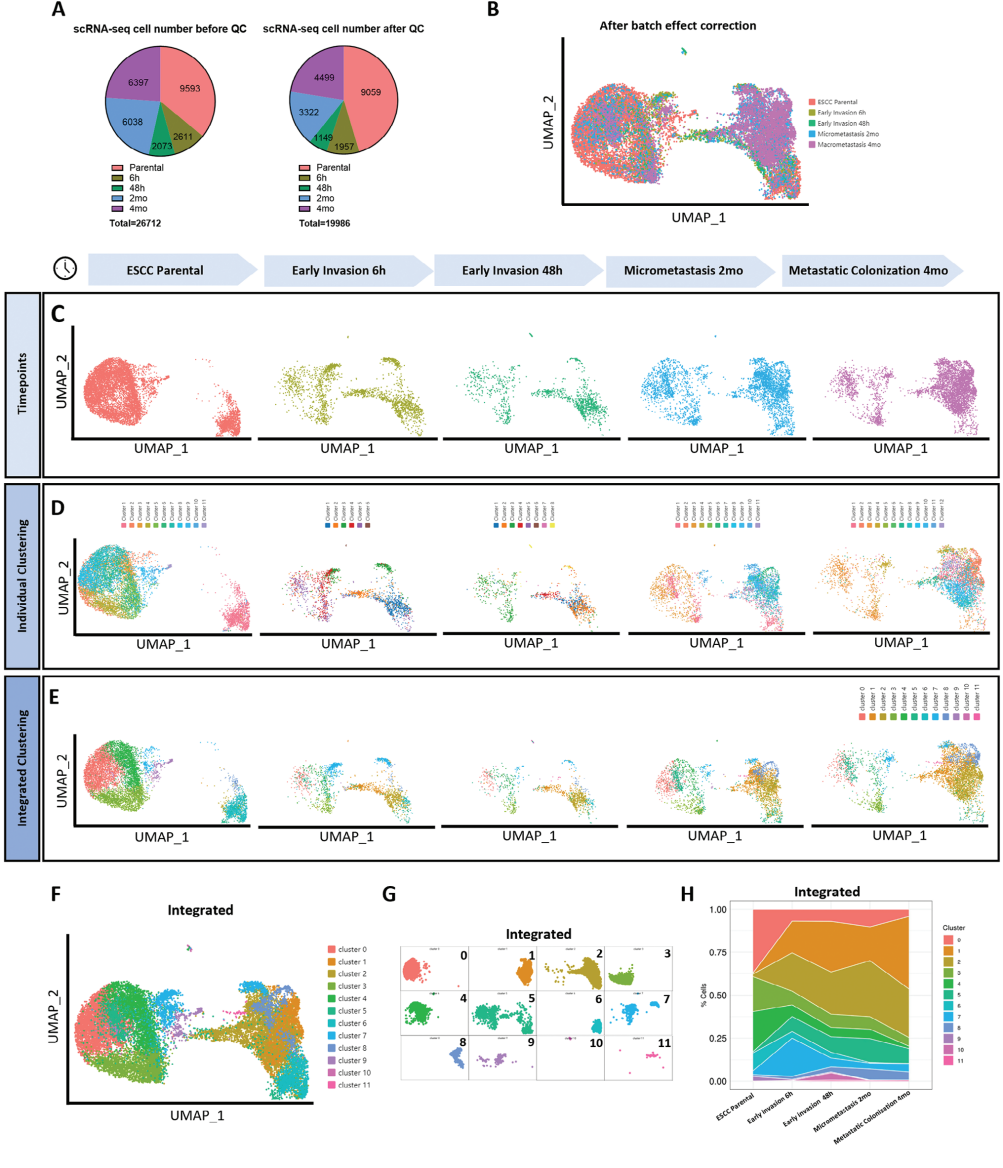
Single‐cell RNA sequencing (scRNA‐seq) analyses and cluster identification. A) Pie charts showing number of cells included before (left) and after (right) quality control (QC) at each timepoint. B) Integrated UMAP plots showing single‐cell spatial distribution after batch effect correction, grouping cells according to timepoints. C) Individual UMAP plots showing single‐cell spatial distribution at corresponding timepoint. D) Individual UMAP plots showing single‐cell spatial distribution of clusters classified within individual timepoints. E) Individual UMAP plots showing single‐cell spatial distribution of clusters classified among integrated cells from all timepoints. F) UMAP plot of overall single‐cell spatial distribution of clusters classified by integration of five timepoints, cluster 0–11. G) UMAP plot of individual single‐cell spatial distribution of clusters classified by integration of five timepoints, cluster 0–11. H) Stacked area chart showing percentage population of cells in each cluster at each timepoint. Five timepoints included parental, early invasion 6 h, early invasion 48 h, micrometastasis 2 mo, and metastatic colonization 4 mo.

To characterize tumor cells at different metastatic stages, GFP positive living tumor cells were flow‐sorted from freshly dissociated mouse lungs at four timepoints (6 h, 48 h, 2 months, and 4 months). By adding parental cells (0 h), 10× scRNA‐seq was performed to study expression profiles of five timepoints. After quality control, 19 986 tumor cells were remained for subsequent bioinformatics analysis (**Figure** **2**A, right). We integrated the five timepoints and corrected for batch effect (Figure 2B). Principal component analysis (PCA) was used for dimensionality reduction, overall (Figure 2B) and each timepoint (Figure 2C) single‐cell spatial distributions were displayed. Notably, three major spatial distribution patterns related to the metastatic stage were shown at five timepoints, including parental stage at 0 h, early metastatic stage at 6 and 48 h, and late metastatic stages at 2 and 4 months. Parental stage cells were mostly distributed in the left half and far lower right corner; early invasion stage cells were scattered in the middle parts and spanning from left to right; and late metastasis stage cells were mostly distributed in the right part of plots.

To check for heterogeneity within timepoints, we next performed clustering classifications within each timepoint (Figure 2D). Interestingly, we found a distinct subpopulation, Parental Cluster 1 (Cluster of Survival: Cluster S), which separated from the rest of Clusters at parental stage (Parental Cluster 2–11). Expression profiling analysis found that Cluster S is not comparable to other clusters at other time points. Next, we performed integrated cluster classification of all cells collected from five timepoints and found a total of 12 integrated clusters (Cluster 0–11) (Figure 2E–G). By collating, Cluster S corresponds to cells from integrated Clusters 1, 2, 5, 6, and 8. We further investigate the integration clusters and discovered dynamic expression pattern along time axis, integrated Clusters 1, 2, 5, 7, 8, and 11 were enriched along the establishment of metastatic colonies (Figure 2H), suggesting Cluster S cells were largely enriched in metastasis subpopulations. These results showed promising evidence that Cluster S cells might favor early survival of metastatic cells to facilitate metastasis initiation, possibly MICs.

### 2.4 Cluster S Represents MICs with Enhanced Metastasizing Features

**Figure 3:**
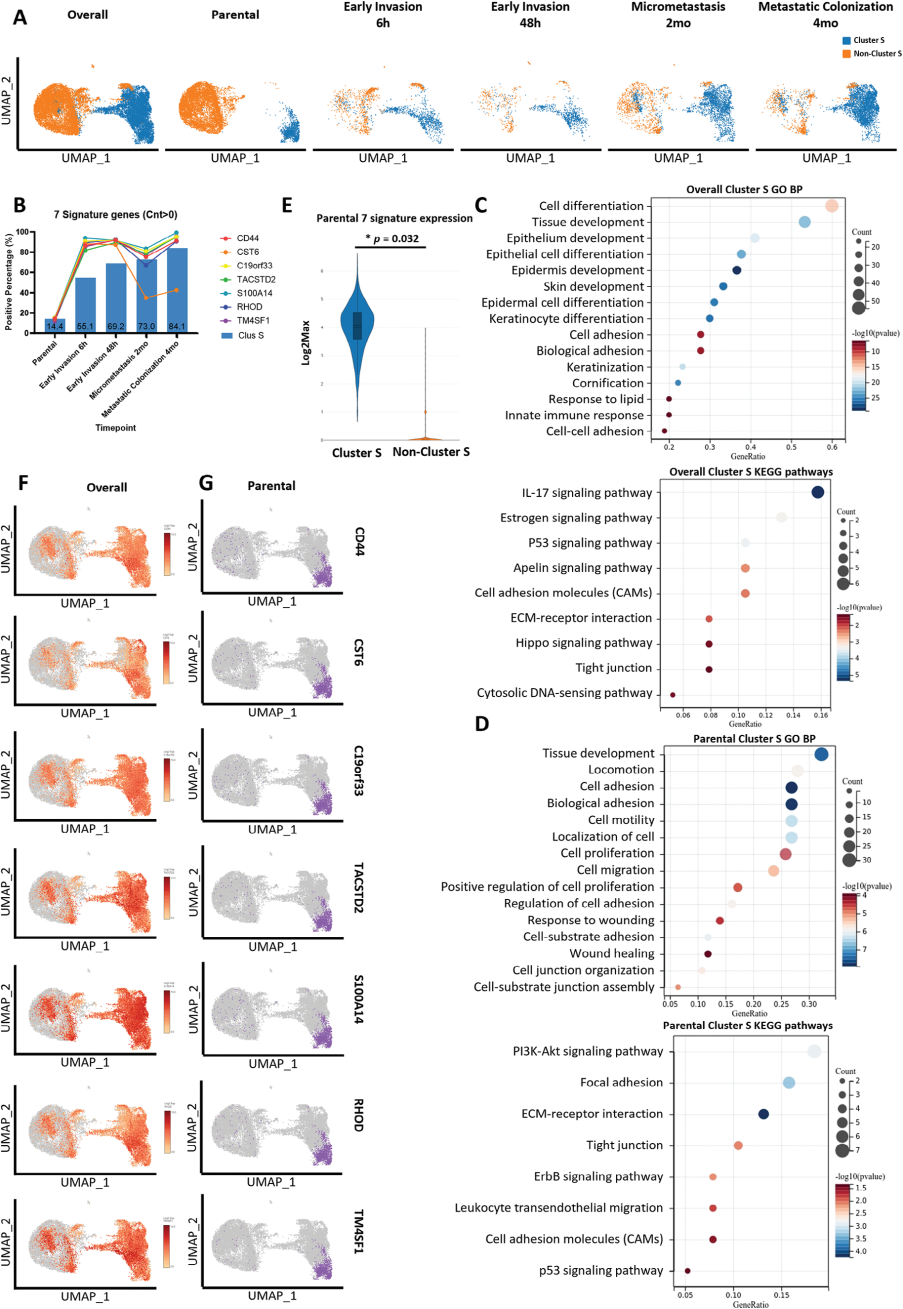
Cluster S spatial distribution and characteristics at different metastatic timepoints. Cluster S (blue) and Non‐cluster S (orange). Timepoints include overall integration, parental, 6 h, 48 h, 2 mo, and 4 mo. Seven metastasis‐initiating signatures (MISs) include CD44, CST6, C19orf33, TACSTD2, S100A14, RHOD, and TM4SF1. A) Individual UMAP plots showing spatial distribution of Cluster S and non‐Cluster S at each timepoints. B) Overlayed bar chart and line graph showing percentage population of cells in Cluster S (bars) and 7 MISs (lines) at each timepoints. C) Dot plots showing biological processes and signaling pathways enriched in Gene ontology (GO; upper) and KEGG (lower) analyses of top 100 significantly upregulated genes in Cluster S at overall integration. D) Dot plots showing biological processes and signaling pathways enriched in Gene ontology (GO; upper) and KEGG (lower) analyses of top 100 significantly upregulated genes in Cluster S at parental stage. E) Violin plot showing 7 MISs expressions in Cluster S and non‐Cluster S at parental stage. Expression defined at Log 2 maximum count. Mann‐Whitney U Test, ****p < 0.0001, ***p < 0.001, **p < 0.01, *p < 0.05, and ns: *p* ≥ 0.05. F) Individual UMAP plots showing spatial distribution of positive cells of each MIS at overall integration. G) Individual UMAP plots showing spatial distribution of positive cells of each MIS at parental stage.

To visualize dynamic change of Cluster S fraction across timepoints, we classified cells into Cluster S and non‐Cluster S (**Figure** **3**A). Most of Cluster S cells (blue) distributed on the right half of UMAP plot and dramatically enriched upon early invasion. Percentage analyses revealed that Cluster S enriched from 14.5% at parental state to more than half population through metastasis with increasing trend (55.1% at 6 h, 84.1% in 4 months, Figure 3B), suggesting that Cluster S cells may be MICs due to their enhanced survival and metastasis capacities.

To evaluate metastatic properties of Cluster S cells, top 100 differentially upregulated genes (*p* < 0.05, log 2 fold‐change >1) of Cluster S were selected for Gene Ontology (GO) and Kyoto Encyclopedia of Genes and Genomes (KEGG) enrichment analysis for overall and individual timepoints (Figure 3C,D; Figure S1A–D, Supporting Information). Results showed that Cluster S from overall integration enhanced cell differentiation and adhesion, increased response to lipid and activated innate immune responses, and upregulating pathways related to cancer immunity (IL‐17,^[^ ^19^ ^]^ estrogen,^[^ ^20^ ^]^ and CAMs signaling),^[^ ^21^ ^]^ tumor metastasis (p53,^[^ ^22^ ^]^ apelin,^[^ ^23^ ^]^ and hippo signaling),^[^ ^24^ ^]^ and angiogenesis (apelin signaling,^[^ ^23^ ^]^ Figure 3C). Cluster S at parental stage has enhanced locomotion, cell adhesion, cell motility, and wound healing, as well as signaling pathways related to tumor migration (PI3K‐Akt,^[^ ^25^ ^]^ ErbB,^[^ ^26^ ^]^ and p53 signaling),^[^ ^22^ ^]^ interaction (ECM‐receptor interaction, CAMs signaling),^[^ ^21^ ^]^ and immunity (leukocyte transendothelial migration; Figure 3D). In summary, we found that Cluster S possesses an integral function in cancer cell migration and cancer‐related immunity, with stage‐specific functions at different timepoints.

### 2.5 Identification of the Potential MIC Signature Genes

To further characterize metastatic properties of Cluster S, we investigated differentially upregulated genes in Cluster S and identified 7 representative genes as potential metastasis‐initiating signature (MIS), including *CD44*, *CST6*, *C19orf33*, *TACSTD2*, *S100A14*, *RHOD*, and *TM4SF1* (see selection criteria in Method). We next examined dynamic population expression patterns of these MIS at each timepoints. Results showed that MIS‐positive cells for all 7 signature genes were mainly detected in Cluster S of parental population (averagely 14%) and were significantly enriched in invasion stages (Figure 3B). We next confirmed that MISs were highly expressed in Cluster S subpopulation at parental stage (Figure 3E). Individual MIS genes showed similar spatial distribution patterns in both ensemble (Figure 3F) and parental stages (Figure 3G), with exception of *CST6* in the ensemble. Since MISs are highly representative of the metastasis initiating Cluster S subpopulation, we hypothesize that expression of these genes is important for maintaining early survival of MICs and facilitating the establishment of pro‐metastasis TME.

### 2.6 MISs 1+1 Signature Filtering Defines MICs at Transcriptional Level

To identify MICs by less stringent simultaneous co‐expression of MISs, MISs 1+1 filter was used to define MICs. The co‐expression of any 1 gene from Group A (C19orf33, S100A14, and RHOD; count ≥1) and any 1 gene from Group B (CST6, CD44, TM4SF1, and TACSTD2; count ≥1) in a single tumor cell was defined as a MIC (Figure S2A, Supporting Information box). Evaluation of MISs 1+1 filter efficiency in parental population demonstrated satisfactory positive predictive value (81.5%), negative predictive value (99.4%), sensitivity (99.5%), specificity (79.9%), and accuracy (89.0%) representing Cluster S subpopulation (Figure S2A,B, Supporting Information). Comparison of the spatial distribution patterns of MISs 1+1 positive cells with any MISs positive cells (Figure S2C, Supporting Information) and Cluster S subpopulation (Figure 3A) at all timepoints verified that similar spatial expression patterns of MISs 1+1 positive cells were highly representative of Cluster S at parental state and those surviving cells at early metastatic states.

### 2.7 MIS‐Enriched (CD44^high^) Cells Possess Metastasis‐Initiating Properties

**Figure 4:**
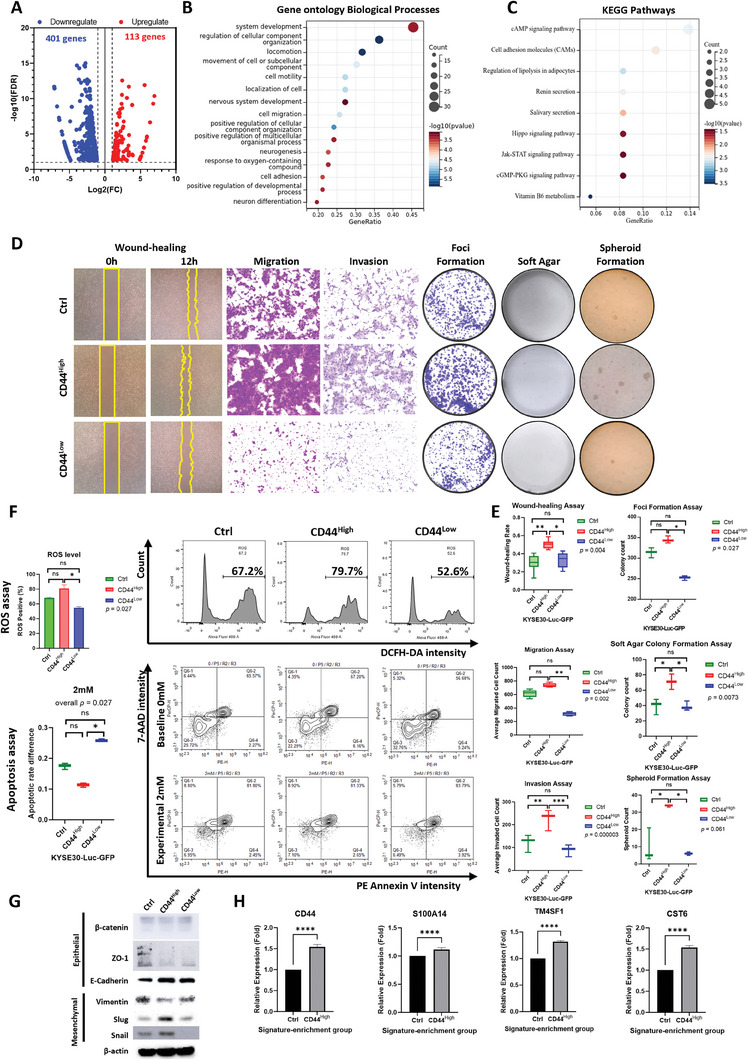
Transcriptomic analyses and functional assays of CD44 high ESCC cells signify metastatic potentials. A) Volcano plot of gene expression of CD44‐signature enriched (CD44 high) KYSE30 cells showing 113 upregulated and 401 downregulated genes (FDR < 0.05, Log2FC ≥ 1) compared to CD44‐signature low (CD44 low) cells. B) Dot plot showing gene ontology (GO) enrichment analyses of top 100 differentially upregulated genes expresses in CD44 high KYSE30 cells, 15 significantly enriched biological processes were shown (FDR < 0.05). C) Dot plot showing KEGG pathway analyses of top 100 differentially upregulated genes expressed in CD44 high KYSE30 cells, 9 significantly enriched pathways were shown (p < 0.05). D) Representative images of wound‐healing, migration, invasion, foci formation, soft agar colony formation, and spheroid formation assays. E) Corresponding statistical analyses results of functional assays. Wound‐healing assay (control *n* = 6, CD44 high *n* = 6, CD44 low *n* = 5); migration assay (control *n* = 5, CD44 high *n* = 5, CD44 low *n* = 5); invasion assay (control *n* = 15, CD44 high *n* = 15, CD44 low *n* = 15); foci formation assay (control *n* = 3, CD44 high *n* = 3, CD44 low *n* = 3); soft agar assay (control *n* = 3, CD44 high *n* = 3, CD44 low *n* = 3); spheroid formation assay (control *n* = 3, CD44 high *n* = 3, CD44 low *n* = 3). Kruskal‐Wallis H Test, ****p < 0.0001, ***p < 0.001, **p < 0.01, *p < 0.05, and ns: p ≥ 0.05. F) Representative images and corresponding statistical analyses results of reactive oxygen species (ROS) assay (upper) and apoptosis assay (lower) of CD44 high (*n* = 3), CD44 low (*n* = 3), and control (n = 3) cells. Kruskal‐Wallis H Test, ****p < 0.0001, ***p < 0.001, **p < 0.01, *p < 0.05, and ns: p ≥ 0.05. G) Western blotting images of protein expressions of epithelial‐mesenchymal transition (EMT) markers of CD44 high, CD44 low, and control cells, including β‐catenin, ZO‐1, E‐cadherin, vimentin, slug, and snail. β‐actin as internal reference. H) Bar charts showing relative RNA expression level of MISs in CD44 high (*n* = 5) and control (*n* = 5) cells by quantitative PCR (qPCR) analyses. Including CD44, S100A14, TM4SF1, and CST6. Mann‐Whitney‐U Test, ****p < 0.0001, ***p < 0.001, **p < 0.01, *p < 0.05, and ns: *p* ≥ 0.05.

To validate potential pro‐surviving and metastasis‐enhancing properties of MICs, MIS‐enriched cells were flow‐sorted by anti‐CD44 antibody (Figure S3A,B, Supporting Information). ScRNA‐seq data from the mice model verified that high population of CD44‐enriched cells is simultaneously co‐expressing other signatures such as C19orf33, TACSTD2, S100A14, RHOD and TM4SF1 in cell cultures and across different metastasis stages and Cluster groups (Figure S4A–D, Supporting Information). Bulk RNA sequencing was applied to compare expression profiles between CD44^high^ and CD44^low^ cells (Table S1, Supporting Information) and 113 differentially upregulated genes were identified in CD44^high^ cells (**Figure** **4**A). GO and KEGG analyses of top 100 upregulated genes revealed CD44^high^ cells are enriched in cell migration, organ development, stress responses, neuron development, and lipid metabolism (Figure 4B,C). These results are consistent with our predictions about the metastatic properties and TME remodeling capacities of MICs, suggesting a potential enhancement of survival and metastatic behavior of MICs.

To test our prediction about metastatic behaviors of MICs, we performed a series of in vitro functional assays on CD44‐enriched and TACSTD2‐enriched subpopulations. Comparing with CD44^low^ cells, CD44^high^ cells exhibited significantly enhanced abilities in wound healing (*p* < 0.05), cell migration (*p* < 0.01), cell invasion (*p* < 0.001), foci formation (anchorage‐dependent, *p* < 0.05), colony formation in soft agar (anchorage‐independent, *p* < 0.05), and spheroid formation (self‐renewal, *p* < 0.05) (Figure 4D,E). We also found that CD44^high^ cells have better anti‐apoptotic ability (*p* < 0.05) despite the increased production of reactive oxidative species (ROS) after oxidative stress stimulation (*p* < 0.05) (Figure 4F). Western blot analysis of epithelial (β‐catenin, ZO‐1, and E‐cadherin) and mesenchymal (vimentin, slug, and snail) markers elucidated the quasi‐epithelial‐mesenchymal (quasi‐EM) state of CD44^high^ cells, having expression of both epithelial and mesenchymal markers simultaneously (Figure 4G). These results confirm the duality of enhanced migration and invasion abilities as well as anchorage‐dependent proliferation and self‐renewal ability of CD44^high^ cells, suggesting that MICs may possess high plasticity in epithelial‐mesenchymal transition (EMT) to adapt to dynamic TME upon stimulation.

Quantitative PCR (qPCR) was used to compare CD44^high^ and CD44^low^ cells to detect expression levels of other three MIS signature markers (*S100A14*, *TM4SF1*, and *CST6*) in CD44^high^ cells. Compared with CD44^low^ cells, results showed that all three markers were significantly upregulated in CD44^high^ cells (Figure 4H), confirming the co‐expression of MISs in MICS at RNA level. In addition, we evaluated functional behaviors of TACSTD2‐enriched cells. Results demonstrated that TACSTD2^high^ cells significantly enhanced wound healing (*p* < 0.01), migration (*p* < 0.001), invasion (*p* < 0.001), foci formation (*p* < 0.05) abilities (Figure S5A,B, Supporting Information). All these results demonstrated an overall enhanced metastatic and proliferative capacity of the MIS‐enriched subpopulations.

### 2.8 MICs Switch from Partial‐Epithelial State to Quasi‐Epithelial‐Mesenchymal State During Early Metastasis Establishment

To address EMT state of MICs during metastasis progress, we evaluated scRNA expression of epithelial marker (CTNNB1, gene for β‐catenin) and mesenchymal marker (SNAI1, gene for Snail) across timepoints (Figure S6A,B, Supporting Information). We defined epithelial (E) state by over 50% population positive (Count>0) of CTNNB1, mesenchymal (M) state by over 10% population positive (Count>0) of SNAI1, and quasi‐epithelial‐mesenchymal (quasi‐EM) state for acquiring both E and M simultaneously based on our data. Our results show that there was a dynamic change of EMT state for cells, from partial‐epithelial (partial‐E) states in parental Cluster S and E state in parental non‐Cluster S to M state at 6 h, quasi‐EM state at 48 h, and returned to E state at 2 months and 4 months. This demonstrated the plasticity of MICs in switching EMT phenotypes upon stimulation by the surrounding environment to adapt and facilitate survival and metastasis colony formation.

### 2.9 MIS‐Enriched Subpopulations have Early‐Survival and Metastatic Colonization Properties

**Figure 5:**
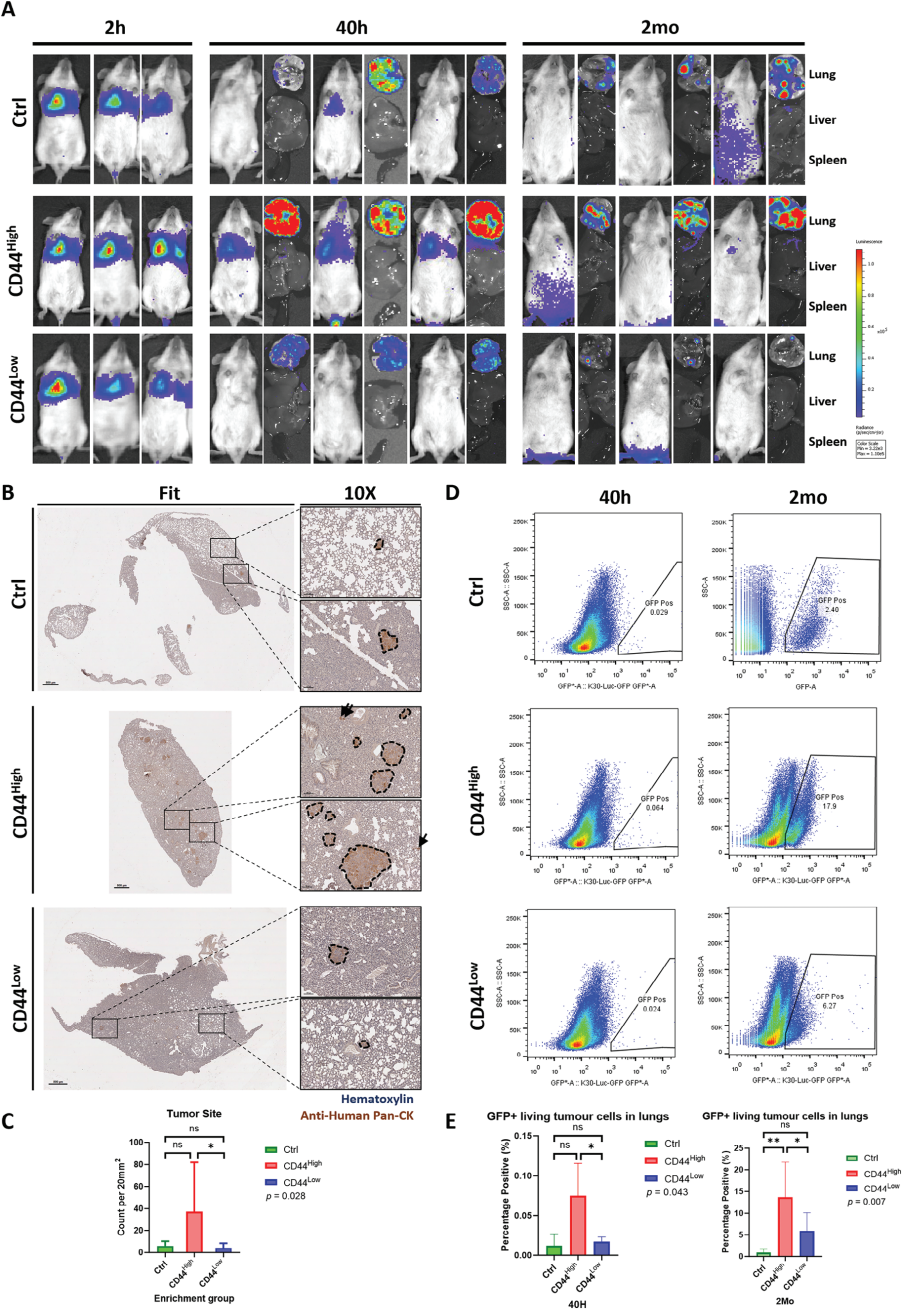
In vitro pulmonary metastasis experiment of CD44 high, low, and control cells in NOD SCID mice. A) Representative in vivo bioluminescence images of mice and corresponding resected tissues (lung, liver, and spleen) with intravenous inoculation of CD44 high, low, and control cells (KYSE30‐Luc‐GFP) at 2 h, 40 h, and 2 mo. Scale: 3.22e3 – 1.10e5 radiance (p/sec/cm^2^/sr). B) Representative IHC staining images of metastasized tumor cells in FFPE mice lungs at 2 mo for CD44 high, low, and control groups. Human pan‐Cytokeratin stained brown. Black arrows and dashed circles indicated tumor cells. Left panel: Whole lung (scale bar = 800 µm). Right small panels: Zoomed‐in sections (scale bar = 100 µm). C) Corresponding tumor site count statistical analyses of IHC staining in CD44 high (*n* = 6), CD44 low (*n* = 5), and control (*n* = 6) groups at 2 mo. D) Representative cell dot plots showing flow cytometry analyses of GFP positive living metastasized tumor cells in each mice lungs of CD44 high, low, and control groups at 40 h (left) and 2 mo (right). E) Corresponding statistical analyses of percentage positive of living metastasized tumor cells in mice lungs of CD44 high (40 h *n* = 3; 2 mo *n* = 6), CD44 low (40 h *n* = 5; 2 mo *n* = 6), and control (40 h *n* = 3; 2 mo *n* = 6) groups at 40 h (left) and 2 mo (right). Kruskal‐Wallis H Test, ****p < 0.0001, ***p < 0.001, **p < 0.01, *p < 0.05, and ns: *p* ≥ 0.05.

To evaluate in vivo metastasis‐initiating properties of MICs, we established a mouse pulmonary metastasis model by intravenously inoculating CD44 or TACSTD2 flow‐sorted MIS‐enriched cells into NOD SCID mice. Bioluminescence signals were monitored from 2 h to 2 months in mice injected with CD44^high^, CD44^low^, and parental KYSE30‐Luc‐GFP. Results found that CD44^high^ group showed the strongest signals in vivo and in freshly resected lungs at 40 h and 2 months compared with CD44^low^ and control groups (**Figure** **5**A), suggesting that an enhanced abilities of MIS‐enriched cells to survive in new niche (40 h) and establish colonization at later stage (2 months). However, a stronger signal only represents more metastatic cells surviving but cannot account for the number and size of colonies formed. Thus, we performed IHC staining to estimate the number and size of colonies formed and flow cytometry analysis to quantify the number of viable cells. IHC staining showed CD44^high^ group formed larger (Figure 5B) and more (*p* < 0.05, Figure 5C) colonies than CD44^low^ and control groups at 2 months, and accelerated formation of visible metastatic nodules (>200 cells) in lungs at 2 months (Figure 5B). Flow cytometry analysis of GFP intensity showed that CD44^high^ group retained more viable tumor cells at both early (40 h, *p* < 0.05) and late metastatic (2 months, *p* < 0.05) stages compared with CD44^low^ group (Figure 5D,E), again echoing the large size nodules we observed in the IHC analysis.

We next investigated the TACSTD2‐enriched subpopulation. Signature co‐expression analyses in scRNA‐seq data validated TACSTD2‐enriched cells have similar expression pattern as CD44‐enriched cells in culture and across different metastasis stages and Cluster groups. (Figure S4E–H, Supporting Information). Similar as CD44^high^ group, TACSTD2^high^ group retained stronger bioluminescence signals in lungs (Figure S5C, Supporting Information) and formed larger sized colonies (Figure S5D, Supporting Information) at 2 months, compared with MIS low groups, though the difference in number of tumor sites formed was not statistically significant (Figure S5E, Supporting Information). Taken together, MIS‐enriched subpopulations possess enhanced in vivo metastatic capacities for early survival and late colonization in lungs.

### 2.10 Detection of MICs using MIS Markers by Multiplex IHC (mIHC)

To test the potential of MISs in MIC detection, mIHC staining was used to detect the co‐expression patterns of 4 selected MISs (CD44, S100A14, RHOD, and TACSTD2) in four ESCC cell lines (KYSE30, KYSE180, KYSE410, and KYSE520). It was found that among four cell lines, ≈10–25% and 20–40% of tumor cells co‐expressed 4 and 3 MISs, respectively (Figure S7A,B, Supporting Information), indicating the prevalence of MIC subpopulation in ESCC.

**Figure 6:**
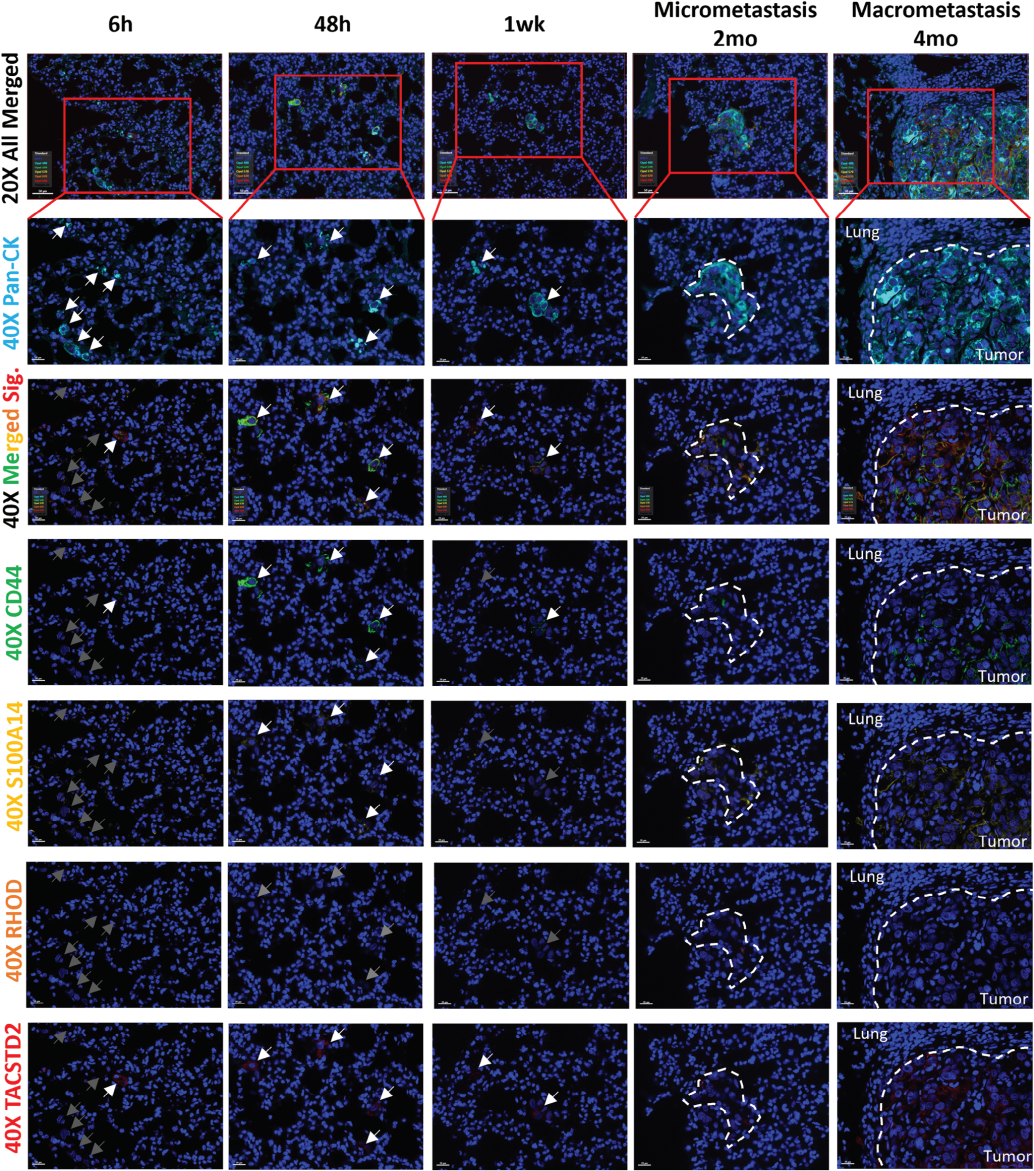
Multiplex IHC (mIHC) staining of MISs in pulmonary metastasized lungs across different timepoints. MISs included CD44 (green; OPAL520), S100A14 (yellow; OPAL570), RHOD (orange; OPAL620), and TACSTD2 (red; OPAL690). Timepoints included 6 h, 48 h, 1wk, 2 mo, and 4 mo. Human pan‐CK (light blue; OPAL480) staining indicates inoculated human ESCC cells, KYSE30‐Luc‐GFP. DAPI (blue) stained nucleus. Arrows and dashed circles indicate tumor cells and nodules, respectively. Grey arrows indicate absence of MIS expression, white arrows, and dashed circles indicate presence of MIS expression in tumor cells or nodules. 20X scale bar: 50 µm. 40X scale bar: 20 µm.

We next introduced mIHC staining into a pulmonary metastasis mouse model to investigate expression pattern of MISs in mouse lungs along the metastatic timeline and elucidate its correlation with tumor cell survival and metastasis in vivo (**Figure** **6**). We found that most single cells did not express MISs 6 h after inoculation, only a few subpopulations displayed co‐expression, which was similar to parental cells. Interestingly, most cells without MIS expression died of survival crisis within 2 days, whereas most surviving colonies co‐expressed MISs at 2 days and 1 week (Figure 6). More MISs co‐expressing tumor cells propagated in metastatic colonies at 2 and 4 months forming larger nodules, suggesting that MISs co‐expression is essential for overcoming early survival crisis and subsequent establishment of metastatic colonization.

### 2.11 MISs Co‐Expression Scores Predict Patient Outcomes

**Figure 7:**
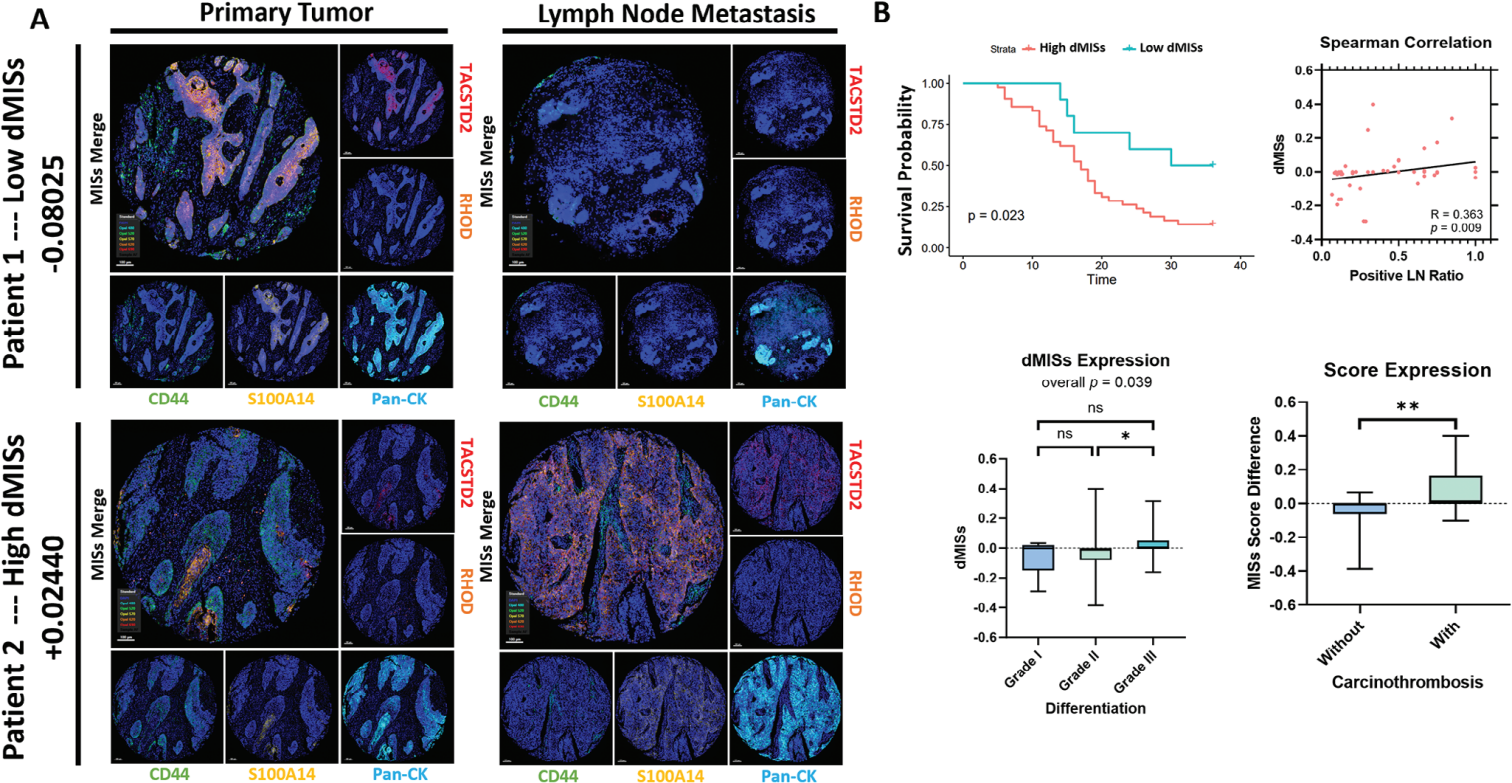
Analyses of differential metastasis‐initiating signature score (dMISs) and patient outcomes. A) Representative multiplex IHC (mIHC) staining of MISs in ESCC tissue microarray (TMA; *n* = 52) with primary tumor (PT) and paired lymph node metastasis (LNM) tissues in two patients representing low (patient 1) and high (patient 2) dMISs. Min dMISs: −0.3851; max dMISs 0.3996; best low dMISs cutoff at ≤ −0.08025 based on R survival package. Low dMISs: *n* = 10; high dMISs: *n* = 42. B) Statistical analyses of dMISs and patient outcomes, including overall survival (OS; top left; *n* = 52), correlation to lymph node metastasis ratio (positive LN ratio; top right; n = 52), tumor differentiation grading (bottom left; *n* = 52), and development of carcinothrombosis (bottom right; n = 52). Cox regression survival curve, Spearman's Rho rank correlation test, Kruskal‐Wallis H test, and Mann‐Whitney U test were performed. ****p < 0.0001, ***p < 0.001, **p < 0.01, *p < 0.05, and ns: *p* ≥ 0.05.

We next examined MIS expression in clinical samples and evaluated the applicability of MIS co‐expression scores in predicting the prognosis of ESCC patients. mIHC staining using 4 selected MISs with high quality antibodies was performed in an ESCC tissue microarray (TMA) containing 244 cases with primary tumors (PT), 57 of which has paired lymph node metastasis (LNM) (**Figure** **7**; Tables S2 and S3, Supporting Information). We first calculated individual signature scores, co‐expressing signature scores for PT and paired LNM nodules, and differential individual and co‐expressing signature scores for each patient comparing PT and LNM scores. We then performed statistical analyses of these scores and corresponding patient outcomes. High scores for single TACSTD2 expression (*p* < 0.01; *p* = 0.039) and S100A14‐TACTSD2 co‐expression (*p* < 0.05; *p* = 0.047) in primary tumors were significantly associated with both presence of LNM (N1 staging) and poorer overall survival (OS) in patients (Figure S8A, Supporting Information), respectively. Comparing LNM with PT, high differential scores of single RHOD expression (R = 0.378, *p* = 0.006; *p* = 0.015), single TACSTD2 expression (R = 0.356, *p* = 0.010; *p* = 0.029), and S100A14‐TACSTD2 co‐expression (R = 0.353, *p* = 0.011; *p* = 0.03) were positively associated with increased LNM ratio (positive LN ratio) and poorer OS (Figure S8B, Supporting Information), respectively. These results demonstrate that the value of individual or dual signature expressions in predicting LNM and OS, although only a portion MISs panel was used.

Therefore, we next formulated a differential MISs score (dMISs) by comparing LNM scores with PT scores, performing co‐expression score calculations using all four MISs, and then analyzed correlations with patient outcomes. We calculated dMISs for each patient and performed Cox regression to classify patients into dMISs high and dMISs low groups (for example, Figure 7A). We found that patients with higher dMISs correlates to poorer OS (*p* = 0.023), increased LNM ratio (positive LN ratio; R = 0.363, *p* = 0.009), higher grade of poorer tumor differentiation (*p* = 0.039), and increased chances to develop carcinothrombosis (*p* < 0.01; Figure 7B). These findings suggest increased co‐expression of MISs in lymph node tissues results in higher dMISs compared with PT and predicts worse patient prognosis.

## 3 Discussion

There is an urging demand to fill the missing early metastatic single‐cell expression profiles in ESCC, especially the identification of MICs and their corresponding expression heterogeneities, to develop a panel of MISs targeting MICs and preventing metastasis. In the present study, we successfully established a multi‐timepoint pulmonary metastasis mouse model to monitor the in vivo metastasis timeline and collected single‐cell transcriptome profiles of living metastatic tumor cells harvested from mouse lungs at different timepoints.

Despite previous theoretical assumptions about metastatic sequential events from invasive cell seeding to metastatic colonization in various cancer types,^[^ ^8^, ^9^, ^12^, ^27^ ^]^ actual demonstration of this sequential pattern experimentally is incomplete. Here, we first visualized the in vivo survival patterns of metastatic cells in lungs from a single colony to metastatic colonization. Next, we identified a dynamic MIC subpopulation (Cluster S) from scRNA‐seq cluster classification and revealed its unique transcriptome. We selected a panel of 7 genes as MIS to represent MICs and developed a MISs 1+1 filter to recognize these cells at transcriptional level. By performing GO and KEGG analyses of scRNA‐seq date from Cluster S and bulk RNA‐seq data from MIS‐enriched subpopulation (CD44^high^), we reconfirmed the metastatic properties and TME remodeling capacities of surviving cells at early metastasis stage, via embryonic‐like tumor implantation and neuro‐immuno‐oncology remodeling approaches. Increasing number of studies have been investigating the role of neuro‐immuno‐oncology interactions as well as neurovascular coupling in cancer progression and metastasis, which raised the importance of involvement of nervous systems and signaling components such as neurotrophic factors, neurotransmitters, and neuroendocrine in shaping the TME.^[^ ^28^ ^]^ Multiple studies in other cancers, such as breast cancer,^[^ ^29^ ^]^ prostate cancer,^[^ ^30^ ^]^ pancreas cancer,^[^ ^31^ ^]^ gastric cancer,^[^ ^32^ ^]^ lung cancer,^[^ ^33^ ^]^ colon and rectum cancer,^[^ ^34^ ^]^ and head and neck cancer,^[^ ^35^ ^]^ have reported the cancer promoting effect of innervation, which refers to the cancer‐associated neural migration and neurogenesis that leads to increased nerve intensity, showing potential positive feedback loops in cancer progression and metastasis.^[^ ^36^ ^]^ However, such studies in ESCC are limited, and currently all of them have been investigated through perineural invasion rather than cancer innervation. Thus, more experimental evidence is needed to verify the role of nervous system in ESCC progression and metastasis.

In terms of the isolation of MISs‐enriched subpopulation, our study has demonstrated through a variety of in vitro functional assays and in vivo metastatic mouse models that the MIS‐enriched subpopulation may represent MICs subpopulation in ESCC with enhanced migration, invasion, proliferation, self‐renewal abilities, and resistances to anoikis, oxidative stress, and apoptosis. We illustrated that MICs are equipped with both early survival and metastasis colonizing abilities to overcome two crises along the metastatic timeline.

Interestingly, we also confirmed the quasi‐EM state of MIS‐enriched subpopulation, expressing both epithelial and mesenchymal markers simultaneously in scRNA‐seq data and cell line western blotting analyses. MIS‐enriched subpopulation demonstrates both epithelial and mesenchymal properties and behaviors via our analyses on functional assays revealing enhanced migrating ability, and metastatic mouse model revealing enhanced anchorage and proliferation abilities. Similar results previously reported in colorectal cancer, breast cancer, prostate cancer, and lung cancer suggest the presence of quasi‐EM phenotypes and spontaneous epithelial‐mesenchymal plasticity (EMP) states, referring to the partial state between complete epithelial and complete mesenchymal transitions (pEMT) in small subpopulations.^[^ ^8^, ^37^, ^38^ ^]^ Such quasi‐epithelial‐mesenchymal‐transition (quasi‐EMT) state could be readily activated to transit toward complete epithelial or mesenchymal states upon stimulation as a switch.^[^ ^8^, ^37^ ^]^ While in this study, we further evidenced the presence of quasi‐EM states of metastatic cells through the multi‐timepoint animal model. There was a dynamic shift of EMT states of MIC (Cluster S) subpopulation from partial‐E state resting in the parental population to M state of in vivo surviving cells at very early metastasis (6 h) and then to quasi‐EM at 48 h, and returning to E state at later metastasis progression (2 months and 4 months). This suggested that MICs are readily transformable from partial‐E state to M and quasi‐EM states upon stimulation from change of microenvironment for better adaptation and establishment of metastasis colonies. The M to quasi‐EM to E state dynamics also revealed sequential involvement of migration and invasion ability at the very beginning of metastasis to move out from the original tumor niche and enter the new niche, which displayed as M state; besides invading, anchorage ability was also required at slightly later stage of early metastasis to settle down, displayed as quasi‐EM state; and finally proliferation ability dominates to colonize at late metastasis stage, displayed as E state. This suggests the high EMP of MICs facilitates the achievement of intrusion of new niche and metastasize at more mesenchymal‐like state, while achieving stationary‐dependent proliferation, TME remodeling, or dormancy entry at more epithelial‐like state. Our findings shed light for future extended investigation of potential detection of quasi‐EMT states in clinical samples and evaluate their predictivity in patient prognosis.

Our study has depicted the specific MICs subpopulation in ESCC via multiplex staining of the simultaneous co‐expression of our MISs panel (i.e., CD44, S100A14, RHOD, and TACSTD2) in cell lines and illustrated the enrichment of MICs in metastatic mice lungs along timeline. We also put forward our MISs to develop differential MIS (dMIS) scores using PT and LNM TMA tissues of ESCC patients to successfully predict poorer patients’ OS, increased LNM ratio, poorer tumor differentiation grade, and increased occurrence of carcinothrombosis with higher dMIS score. Of note, the intravenous injection‐based metastasis mouse model mimics the presence of tumor cells in the circulation and allows evasion of vessels which is partially similar to that in patients with carcinothrombosis, which has a higher chance of shedding tumor cells along the circulation and finally evades from bloodstream and settle at the distal organ. Though the model was based on blood vessel metastasis that is different from lymph vessels, dMIS score successfully predicts both carcinothrombosis and lymph node metastasis, patients with carcinothrombosis have significantly higher dMIS, and high dMIS correlates to an increased number of tumor‐positive lymph node detected. These demonstrated the potential common involvement of the signature genes in intravasation and extravasation processes of both blood and lymph vessels. Transferable clinical applications of our MIS panel could aid diagnosis, adjust therapeutic treatments and improve awareness of the potential development of metastasis and vascular obstruction.

## 4 Limitations of the Study

Mouse model used in this study was immune compromised NOD SCID mice with human ESCC cells inoculated to characterize genes upregulated in human ESCC MICs. The adapted immune system is compromised, B cells and T cells were present but non‐functional. Therefore, only the effect of innate immune system on tumor cells could be examined, while investigation in adapted immune system was not applicable in this study. Further validations in immune competent mice with the proposed signature panel from this study would confirm the effect of the adaptive immune system on the MICs. The scRNA‐seq utilized solely tumor cells retrieved from different metastatic stages at a multi‐timepoint manner, the direct cell‐cell interaction with the surrounding TME could not be investigated, interaction and response to surrounding TME was only investigated through identification of differentially expressed genes, GO enrichment of biological process, and KEGG pathway analyses. Future investigation could be done to include both early metastasizing ESCC cells and surrounding cells in the TME to provide a more in depth examination in MIC‐TME early metastasis interactions.

## 5 Conclusion

In summary, our study first successfully embodied the stage‐specific expression profiles of ESCC cells along the metastatic progression timeline to fill in the missing puzzles, identified and verified specific quasi‐mesenchymal MICs subpopulations with a MIS panel and revealed the potential early‐stage metastasis neuro‐immuno‐oncological TME remodeling approaches that facilitates metastasis colonization. We also utilized this MIS panel to predict ESCC patient outcomes and look forward to further refining it to clinical practice.

## Conflict of Interest

The authors declare no conflict of interest.

## Author Contributions

C.N.W. and Y.Z. contributed equally to this work as co‐first author. C.N.W. conceived the study, coordinated, and performed the experiments, conducted data analyses and bioinformatics analyses, and wrote the manuscript. Y.Z. performed related experiments. B.R. conducted scRNA‐seq data analyses. S.W. assisted in related experiments and data analyses. H.Z. assisted data analyses. J.L. assisted in related experiments. Y.L. assisted data analyses. Y.Q. provided clinical samples. P.J. assisted data analyses. V.H.‐F.L. supervised the study. X.‐Y.G conceived and supervised the study. All the authors have read and approved the manuscript.

## Supporting information

Supporting Information

## Data Availability

The data that support the findings of this study are openly available in NCBI GEO at https://www.ncbi.nlm.nih.gov/geo/query/acc.cgi?acc=GSE249058, reference number 249058.
