# Supplementary material for: Identification and Characterization of Metastasis‐Initiating Cells in ESCC in a Multi‐Timepoint Pulmonary Metastasis Mouse Model
Source: Adv Sci (Weinh). 2024 Jun 12;11(30):2401590. doi: 10.1002/advs.202401590 (PMC11321633; doi:10.1002/advs.202401590)
Supplement: Supplementary file 1 — Supporting Information [file ADVS-11-2401590-s001.docx]

**Supplementary Appendix**

**Supplementary Tables:**

**Table S1. Bulk RNA sequencing statistics on CD44 high and CD44 low cells.**

| **Sample Name** | **Number of raw reads (Read1+Read2)** | **% bases of ≥Q30** | **Number of filtered reads** | **% of filtered reads** | **Number of reads mapped to reference genome** | **% of reads mapped to reference genome** |
| --- | --- | --- | --- | --- | --- | --- |
| KYSE30-CD44_Low | 64,282,568 | 93% | 62,642,438 | 97.45% | 57,304,460 | 91.49% |
| KYSE30-CD44_High | 59,528,688 | 93% | 58,007,230 | 97.44% | 53,404,770 | 92.07% |

**Table S2. Clinicopathological characteristics of ESCC patients enrolled for tissue microarray analyses. Histological classifications referred to 5th edition AJCC Cancer Staging Manual.**

| **Group** | | **Overall** |
| --- | --- | --- |
| **n** | | 244 |
| **Age (mean (SD))** | | 60.03 (9.27) |
| **Gender (%)** | **Female** | 104 (42.6) |
|  | **Male** | 140 (57.4) |
| **Site (%)** | **Cervical** | 1 ( 0.4) |
|  | **Upper** | 51 (21.2) |
|  | **Middle** | 162 (67.2) |
|  | **Lower** | 20 ( 8.3) |
|  | **Multiple** | 7 ( 2.9) |
|  | **Unknown** | 3（1.2） |
| **Grade (%)** | **1** | 27 (11.1) |
|  | **2** | 157 (64.3) |
|  | **3** | 58 (23.8) |
|  | **4** | 0(0) |
|  | **Unknown** | 2（0.8） |
| **Stage (%)** | **0** | 3 ( 1.2) |
|  | **I** | 9 ( 3.7) |
|  | **IIA/IIB** | 153 (63.5) |
|  | **III** | 74 (30.7) |
|  | **IV/IVA/IVB** | 5 ( 2.1) |
| **T (%)** | **In situ** | 3 (1.2) |
|  | **1** | 11 ( 4.5) |
|  | **2** | 77 (31.6) |
|  | **3** | 152 (62.3) |
|  | **4** | 1 ( 0.4) |
| **N (%)** | **0** | 142 (58.2) |
|  | **1** | 102 (41.8) |
| **M (%)** | **0** | 239 (98.0) |
|  | **1** | 5 ( 2.0) |
| **Carcinothrombosis (%)** | **Without** | 194 (79.5) |
|  | **With** | 50 (20.5) |
| **Perineural invasion (%)** | **Without** | 195 (79.9) |
|  | **With** | 49 (20.1) |
| **Survival Month (median [IQR])** | | 21.50 [15.00, 36.00] |

**Table S3. Clinicopathological characteristics of ESCC patients with paired metastatic lymph nodes enrolled for tissue microarray analyses. Histological classifications referred to 5th edition AJCC Cancer Staging Manual.**

| **Group** | | **Overall** |
| --- | --- | --- |
| **n** | | 57 |
| **Age (mean (SD))** | | 59.7 (9.4) |
| **Gender (%)** | **Female** | 22 (38.6) |
|  | **Male** | 35 (61.4) |
| **Site (%)** | **Cervical** | 0(0) |
|  | **Upper** | 4 (7) |
|  | **Middle** | 43 (75.4) |
|  | **Lower** | 6 (10.5) |
|  | **Multiple** | 3 (5.3) |
|  | **Unknown** | 1（1.8） |
| **Grade (%)** | **1** | 3 (5.3) |
|  | **2** | 31 (54.4) |
|  | **3** | 21 (36.8) |
|  | **4** | 0 (0) |
|  | **Unknown** | 2 (3.5) |
| **Stage (%)** | **0** | 0 (0) |
|  | **I** | 0 (0) |
|  | **IIA/IIB** | 19 (33.3) |
|  | **III** | 32 (56.1) |
|  | **IV/IVA/IVB** | 6 (10.5) |
| **T (%)** | **In situ** | 0 (0) |
|  | **1** | 2 (3.5) |
|  | **2** | 18 (31.6) |
|  | **3** | 37 (64.9) |
|  | **4** | 0 (0) |
| **N (%)** | **0** | 0 (0) |
|  | **1** | 57 (100) |
| **M (%)** | **0** | 51 (89.5) |
|  | **1** | 6 (10.5) |
| **Carcinothrombosis (%)** | **Without** | 39 (68.4) |
|  | **With** | 18 (31.6) |
| **Perineural invasion (%)** | **Without** | 42 (73.7) |
|  | **With** | 15 (26.3) |
| **Survival Month (median [IQR])** | | 18 [12.5, 30.5] |

**Table S4. Primer sequences used for qPCR.**

| **Gene** | **Primer sequence** |
| --- | --- |
| **CD44** | Forward 5’-CTGATGATGACGTGAGCAGC-3’  Reverse 5’-ACTGTCTTCGTCTGGGATGG-3’ |
| **S100A14** | Forward 5’-TTCAGTGATGTGGAGAGGGC-3’  Reverse 5’-TTCTCTTCCAGGCCACAGTT-3’ |
| **TM4SF1** | Forward 5’-CGTGTGGTTCTTTTCTGGCA-3’  Reverse 5’-CCACAGTTTTCATGGCCACA-3’ |
| **CST6** | Forward 5’-TACTTCCGAGACACGCACAT-3’  Reverse 5’-TCAAAGTCACAGCGCAGCTT-3’ |

**Supplementary Figures:**


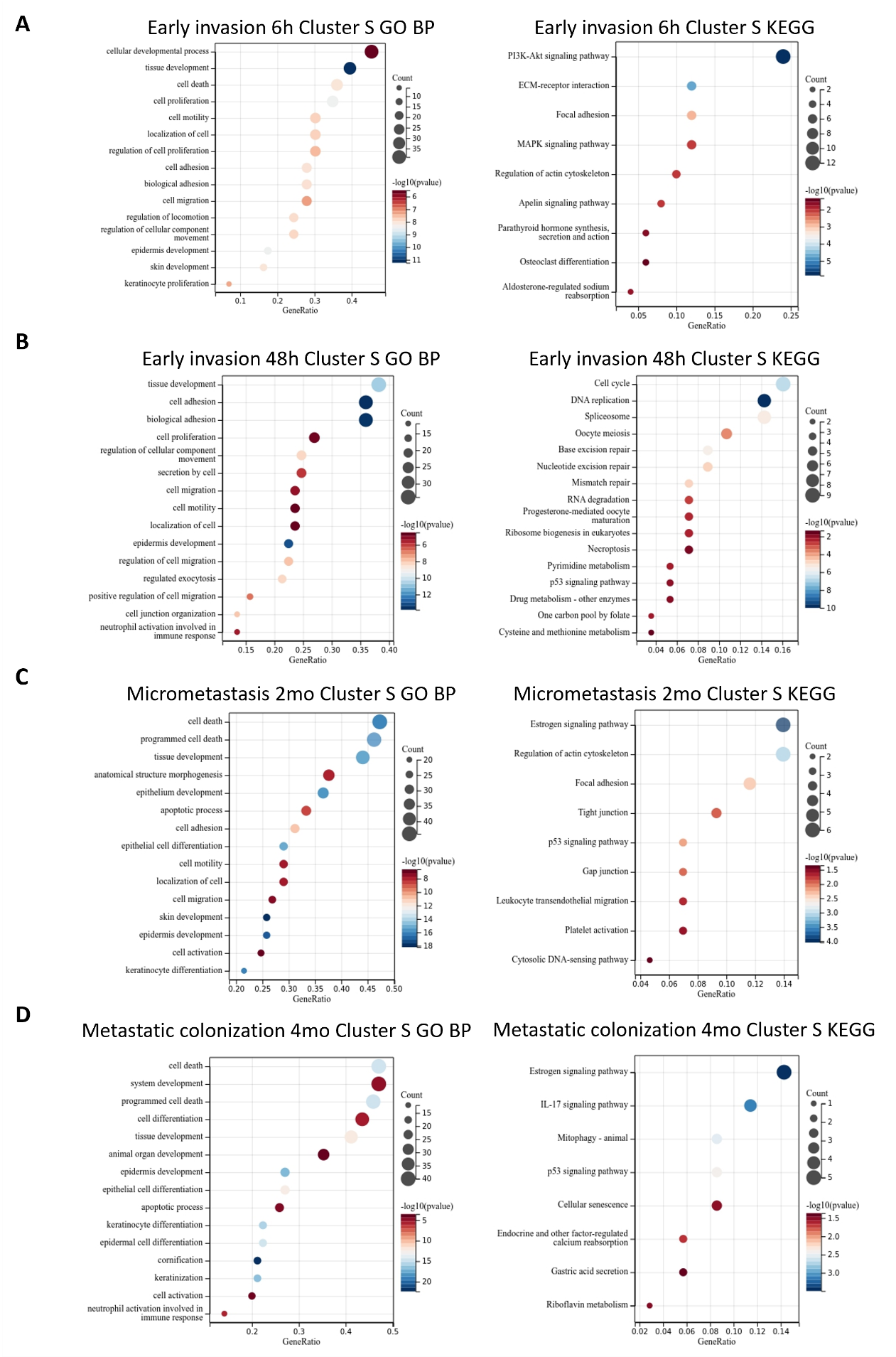


**Figure S1. Gene ontology and KEGG enrichment analysis of top 100 differentially upregulated genes of Cluster S at each timepoints.**

A) Early invasion at 6h.

B) Early invasion at 48h.

C) Micrometastasis at 2mo.

D) Metastatic colonization at 4mo.

Gene ontology biological processes on the left, KEGG enrichment analysis on the right.


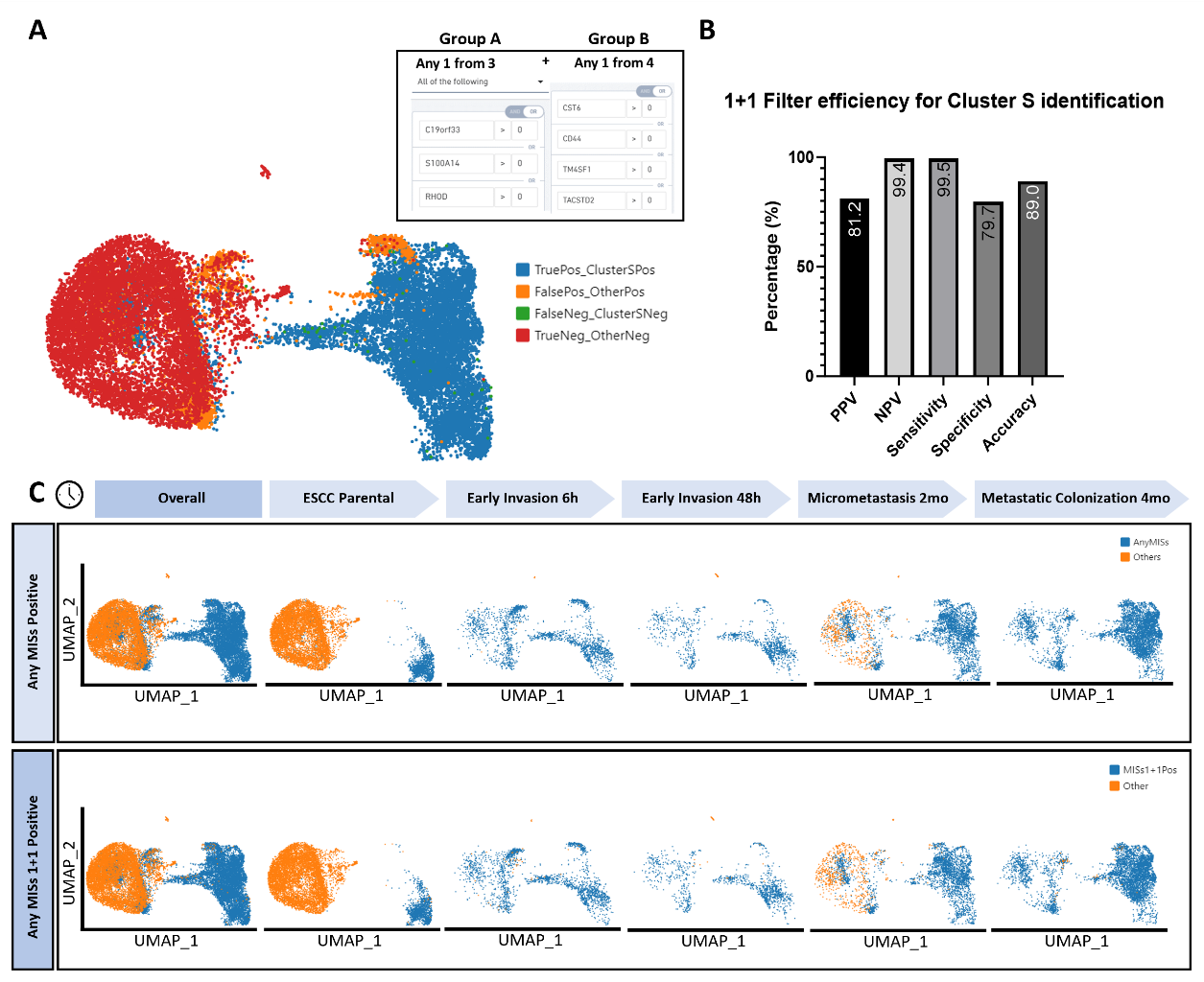


**Figure S2. MISs 1+1 filter efficiency evaluation.**

A) UMAP plot showing spatial distribution of Cluster S measurement using MISs 1+1 filter, including true positive (TruePos_ClusterSPos; blue), false positive (FalsePos_OtherPos; orange), false negative (FalseNeg_ClusterSNeg; green), and true negative (TrueNeg_OtherNeg; red). The right box indicates combinations for MISs 1+1 filter, any 1 from 3 genes in Group A including C19orf33, S100A14, and RHOD, plus any 1 from 4 genes in Group B including CST6, CD44, TM4SF1, and TACSTD2. Positive expression defined by count >0.

B) Bar chart showing efficiency of MISs 1+1 filter for Cluster S identification, including positive predictive value (PPV), negative predictive value (NPV), sensitivity, specificity, and accuracy. Refer to method section for efficiency calculation.

C) Individual UMAP plots showing positive cells (blue) and negative cells (orange) of original any MISs positive definition (upper panel) and refined any MISs 1+1 positive filter (lower panel) at each timepoints, including overall integration, parental, 6h, 48h, 2mo, and 4mo. Positive expression defined by count >0.


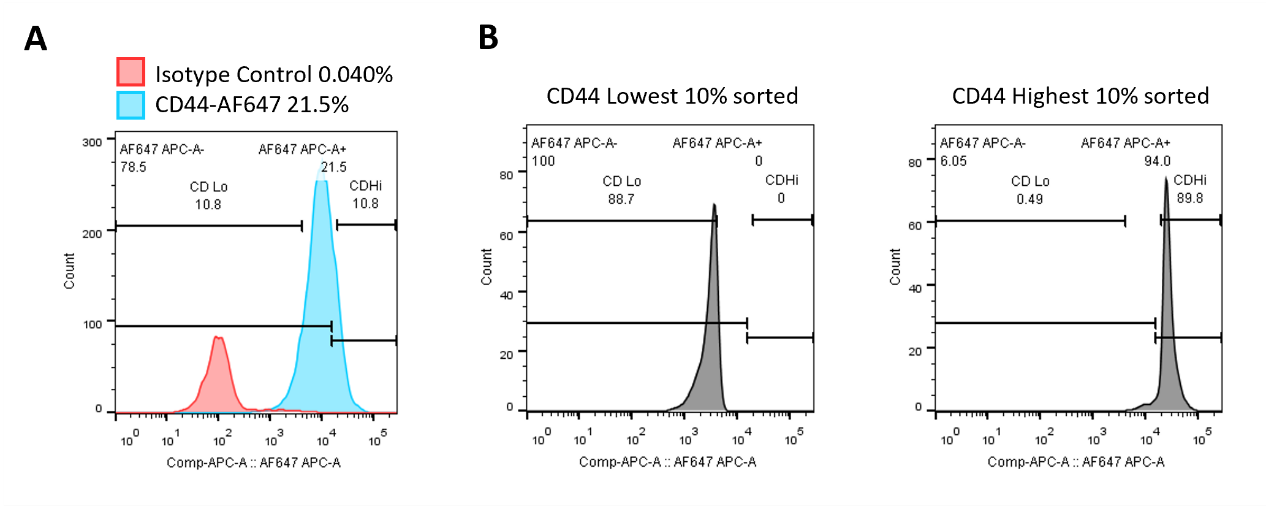


**Figure S3. Flow cytometry fluorescent-activated cell sorting (FACS) of CD44-enriched cells.**

A) Histogram showing gating of stained cells by isotype control.

B) Histograms showing reloading of sorted CD44 low (left) and CD44 high (right) cells.


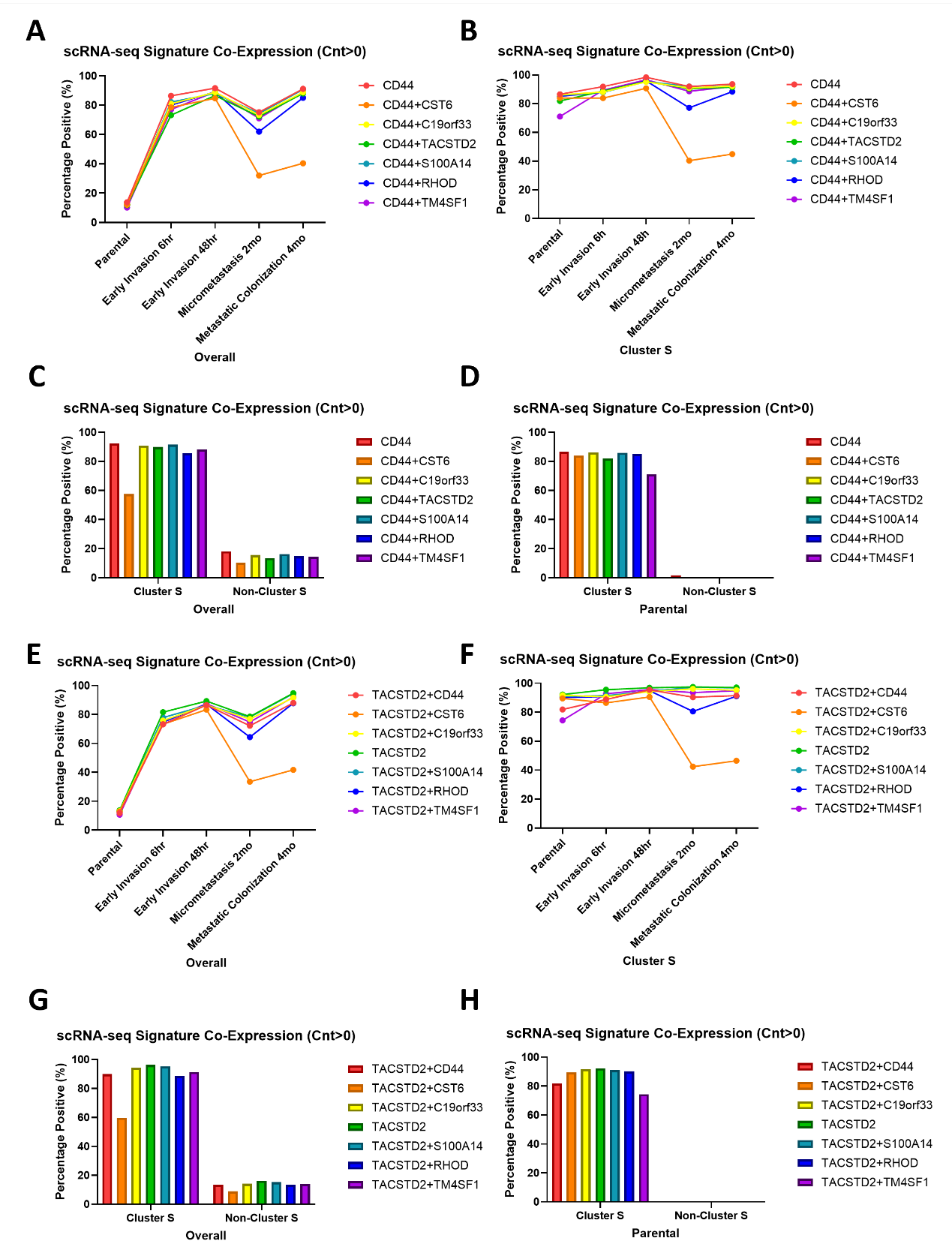


**Figure S4. ScRNA-seq analyses of co-expression of enriched-MIS and other six signatures (Count>0).**

Percentage positive population of cells under single filter for CD44 or TACSTD2, co-expression filter for CD44 and other signatures including CST6, C19orf33, TACSTD2, S100A14, RHOD and TM4SF1 or co-expression filter for TACSTD2 and other signatures including CD44, CST6, C19orf33, S100A14, RHOD and TM4SF1.

A) Line graph showing signature co-expressions across 5 timepoints for CD44 filters.

B) Line graph showing signature co-expressions across 5 timepoints in Cluster S subpopulation for CD44 filters.

C) Bar chart showing signature co-expressions in overall Cluster S and non-Cluster S subpopulations for CD44 filters.

D) Bar chart showing signature co-expressions in parental Cluster S and non-Cluster S subpopulations for CD44 filters.

E) Line graph showing signature co-expressions across 5 timepoints for TACSTD2 filters.

F) Line graph showing signature co-expressions across 5 timepoints in Cluster S subpopulation for TACSTD2 filters.

G) Bar chart showing signature co-expressions in overall Cluster S and non-Cluster S subpopulations for TACSTD2 filters.

H) Bar chart showing signature co-expressions in parental Cluster S and non-Cluster S subpopulations for TACSTD2 filters.


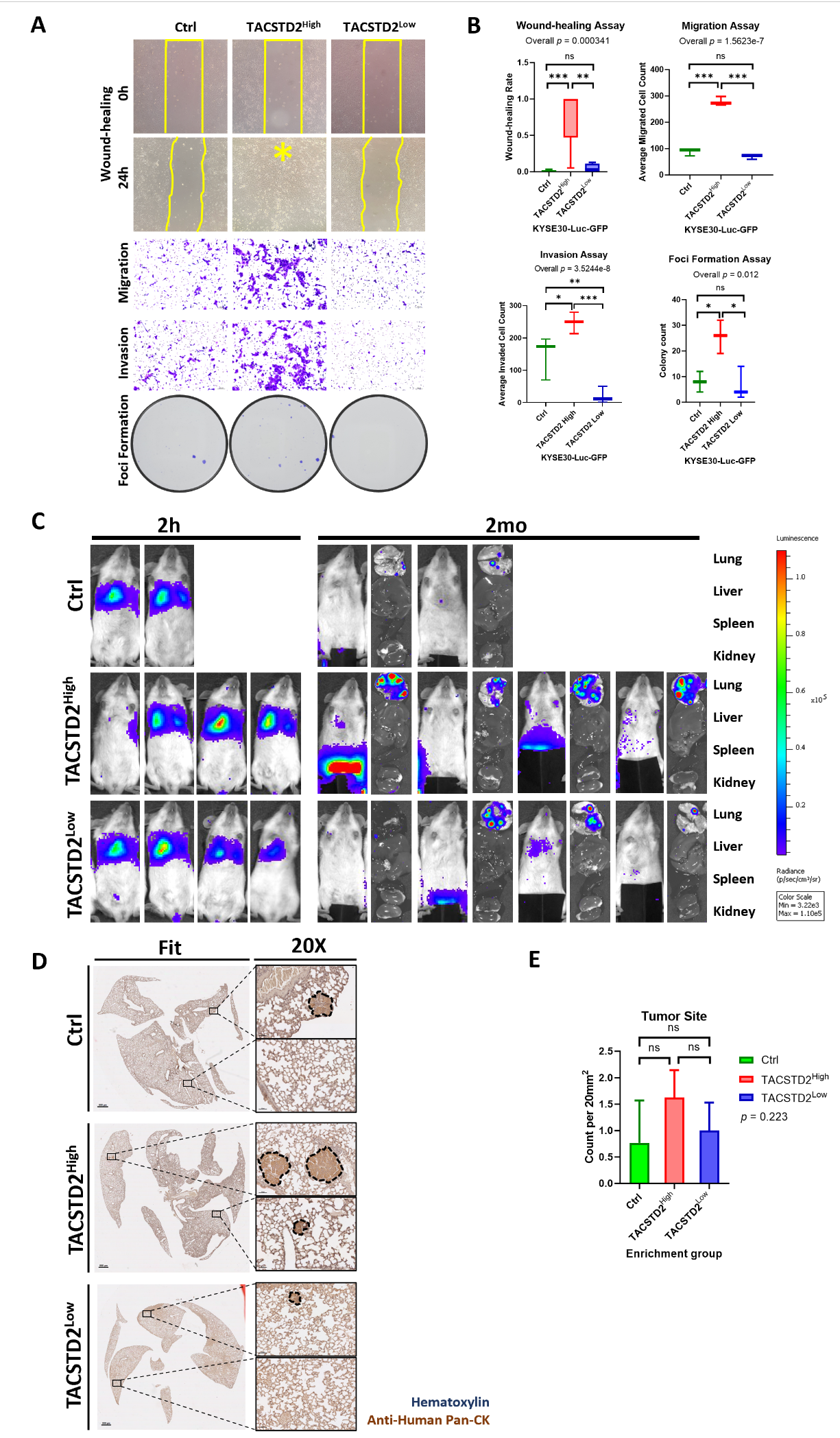


**Figure S5.** **Functional assays and in vivo pulmonary metastasis experiments results of TACSTD2 high, low and control cells, KYSE30-Luc-GFP.**

A) Representative images of wound-healing, migration, invasion, and foci formation assays.

B) Corresponding statistical analyses results of functional assays. Wound-healing assay (control n=6, TACSTD2 high n=5, TACSTD2 low n=4), migration assay (control n=15, TACSTD2 high n=15, TACSTD2 low n=15), invasion assay (control n=15, TACSTD2 high n=15, TACSTD2 low n=15), and foci formation assay (control n=3, TACSTD2 high n=3, TACSTD2 low n=3).

C) Representative in vivo bioluminescence images of mice and corresponding resected tissues (lung, liver, spleen, and kidney) with intravenous inoculation of TACSTD2 high, low, and control cells (KYSE30-Luc-GFP) at 2h and 2mo. Scale: 3.22e3 – 1.10e5 radiance (p/sec/cm^2^/sr).

D) Representative IHC staining images of metastasized tumor cells in FFPE mice lungs at 2mo for TACSTD2 high, low, and control groups. IHC staining with rabbit anti-human pan-Cytokeratin antibody (ab217916) at 1:400 dilution, with prior heat-induced epitope retrieval (HIER) at 95°C for 20 minutes in DAKO citrate target antigen retrieval buffer (pH6; S1699). Black dashed circles indicated tumor colonies. Left panel: Whole lung (scale bar = 800µm). Right small panels: Zoomed-in sections (scale bar = 50µm).

E) Corresponding tumor site count statistical analyses of IHC staining in TACSTD2 high (n=4), low (n=3), and control (n=3) groups at 2mo.

Kruskal-Wallis H Test, ****: p<0.0001, ***: p<0.001, **: p<0.01, *: p<0.05, and ns: p≥0.05.

***
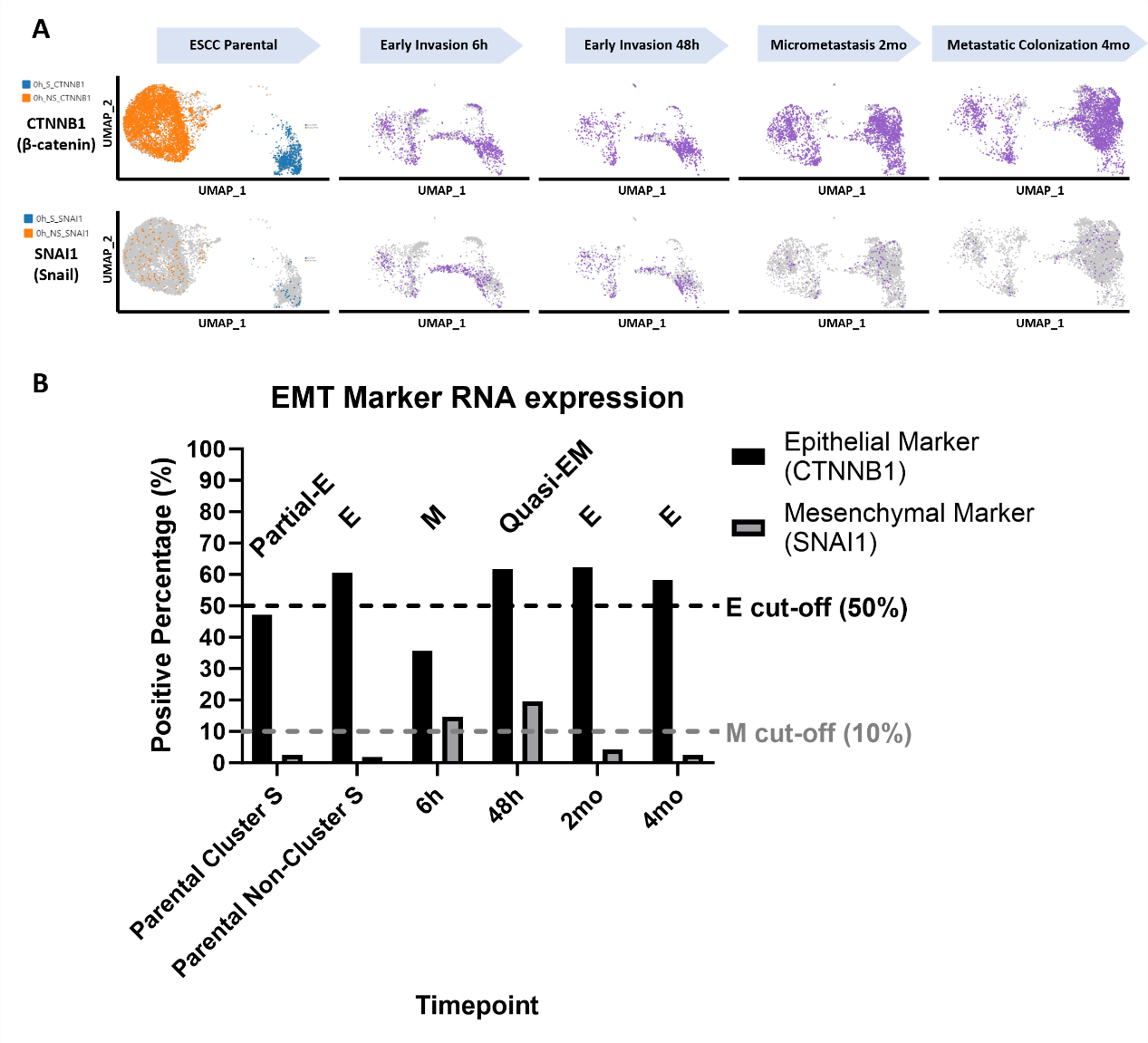
***

**Figure S6.** **Epithelial-mesenchymal-transition (EMT) markers RNA expressions across timepoints.**

A) UMAP plots showing distributions of cells with or without RNA expressions of EMT markers across timepoints. Upper panel: CTNNB1 (coding gene for β-catenin protein, epithelial marker); lower panel: SNAI1 (coding gene for Snail protein, mesenchymal marker). Timepoints include parental, 6h, 48h, 2mo, and 4mo. Positive expressing cells are indicated in blue (parental Cluster S), orange (parental non-Cluster S), and purple (6h, 48h, 2mo, and 4mo). Negative cells are indicated in grey (all timepoints).

B) Bar chart showing positive percentage of cell population expressing the EMT markers at different timepoints. Population and timepoints include parental Cluster S, parental non-Cluster S, 6h, 48h, 2mo, and 4mo. Epithelial marker is represented by CTNNB1, mesenchymal marker is represented by SNAI1. EMT states include epithelial (E), partial-epithelial (partial-E), quasi-epithelial-mesenchymal (quasi-EM), and mesenchymal (M). Epithelial phenotype cutoff at 50%, mesenchymal phenotype cutoff at 10%.


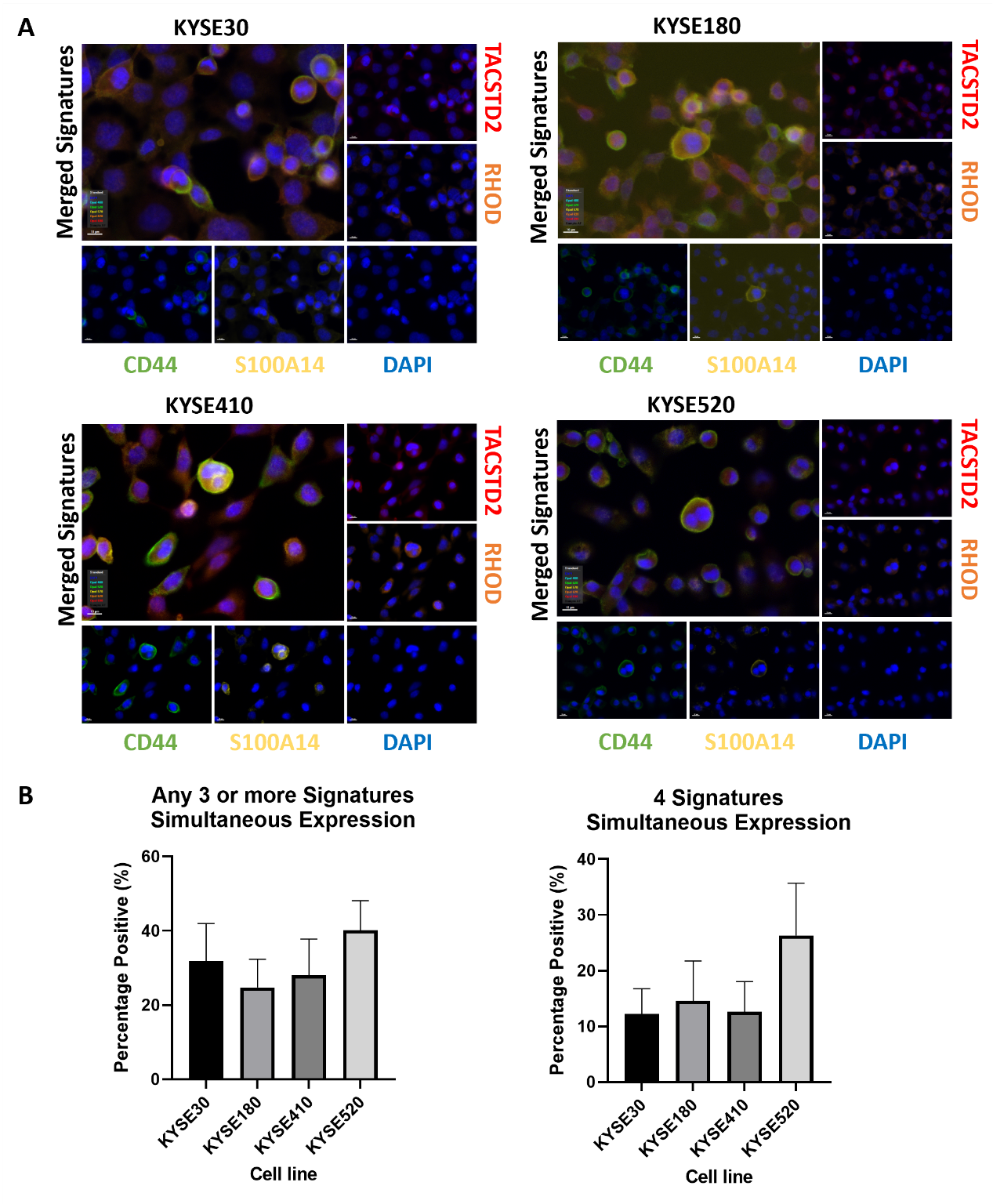


**Figure S7.** **Multiplex IHC (mIHC) staining of MISs in different ESCC cell lines.**

A) Representative images of mIHC staining in KYSE30 (top left), KYSE180 (top right), KYSE410 (bottom left), and KYSE520 (bottom right). MISs included CD44 (green; OPAL520), S100A14 (yellow; OPAL570), RHOD (orange; OPAL620), and TACSTD2 (red; OPAL690). DAPI (blue) was stained to indicate cell nucleus.

B) Bar charts showing corresponding percentage positive population with any 3 or more MISs (left) and all 4 MISs (right) simultaneous expression in each cell lines, e.g. KYSE30 (3sig n=5; 3sig n=5); KYSE180 (3sig n=5; 3sig n=5); KYSE410 (3sig n=5; 3sig n=5); KYSE520 (3sig n=5; 3sig n=5).


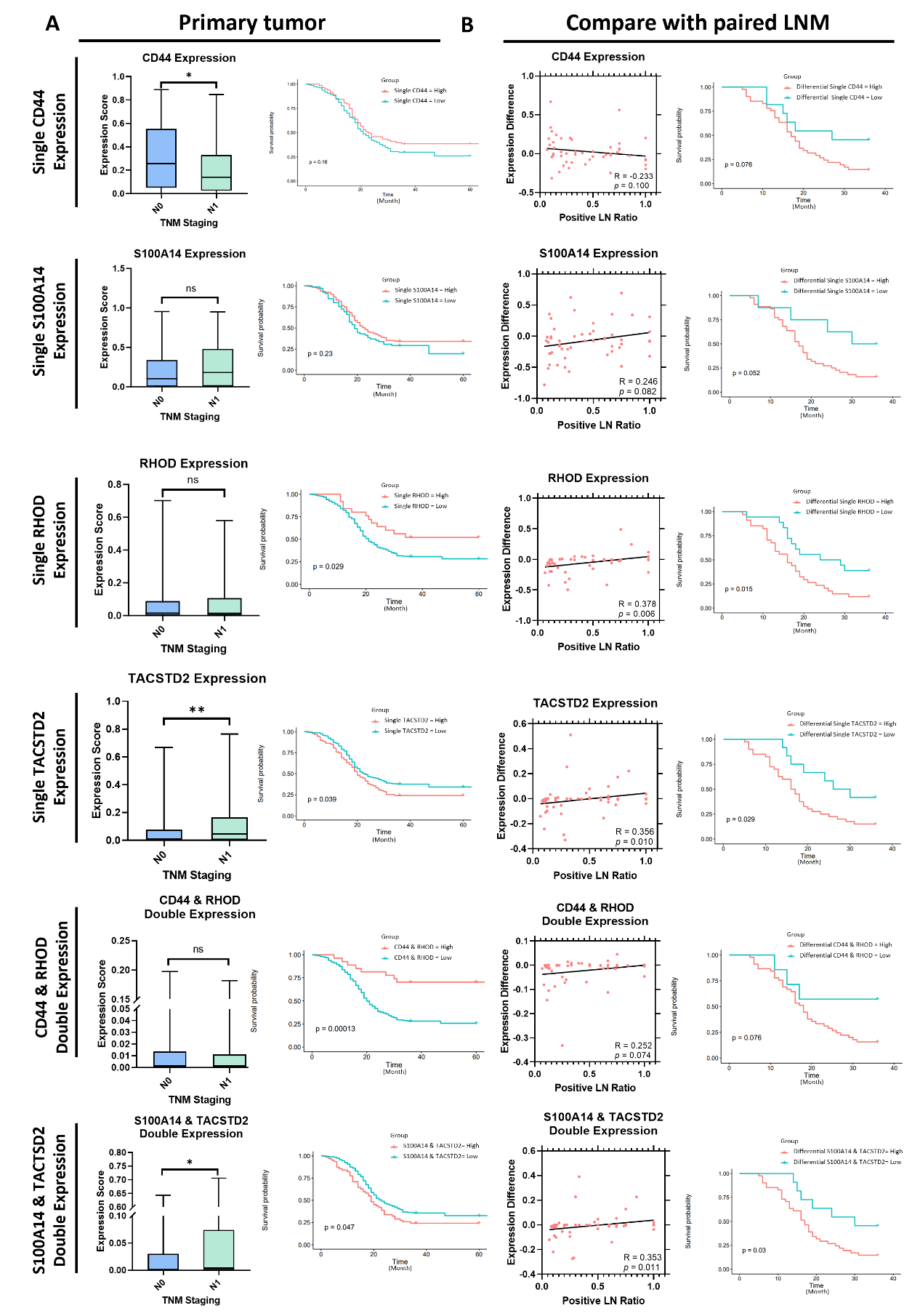


**Figure S8.** **Individual signature and co-expression tumor score and differential scores in ESCC tissue microarray (TMA).**

A) Analyses of signature scores in primary tumor (PT) and TNM lymph node staging and overall survival (OS) in patients. Scores include single expression of CD44, S100A14, RHOD, and TACTSD2, and co-expression of CD44-RHOD and S100A14-TACSTD2. TNM staging (N0 n=142), N1 n=102); single CD44 (high n=83, low n=162); single S100A14 (high n=179, low n=65); single RHOD (high n=25, low n=219); single TACSTD2 (high n=87, low n=157); double CD44 and RHOD (high n=27, low n=217); double S100A14 and TACSTD2 (high n=62, low n=182).

B) Analyses of differential signature scores calculated by comparing PT and lymph node metastasis (LNM) tissues, and LNM ratio (positive LN ratio) and OS. Differential scores include single expression of CD44 (n=52), S100A14 (n=52), RHOD (n=52), and TACTSD2 (n=52), and co-expression of CD44-RHOD (n=52) and S100A14-TACSTD2 (n=52). Differential single CD44 (high n=41, low n=11), differential single S100A14 (high n=44, low n=8), differential single RHOD (high n=34, low n=18), differential single TACSTD2 (high n=40, low n=12), differential CD44 and RHOD (high n=45, low n=7), differential S100A14 and TACSTD2 (high n=41, low n=11).

Mann-Whitney U test, Spearman’s Rho rank correlation test, and Cox regression survival curve were performed. ****: p<0.0001, ***: p<0.001, **: p<0.01, *: p<0.05, and ns: p≥0.05.

**Experimental Section:**

**Cell lines and cell culture**

Human ESCC cell-lines (KYSE30, KYSE180, KYSE410, KYSE520) were cultured in RPMI1640 medium with L-glutamine (Gibco, NY) supplemented with 10% v/v fetal bovine serum (Gibco, NY) and 1% v/v penicillin-streptomycin. Cells were incubated at 37°C humidified cell chamber with 5% CO_2_ supply. Luciferase (Luc) and green fluorescence protein (GFP) labelling was established via lentiviral transduction, followed by drug selection, including blasticidin (0.014mg/mL, InvivoGen, San Diego) and puromycin dihydrochloride (0.001mg/mL, Gibco, NY), respectively.

**Tissue microarray (TMA)**

A total of 244 pairs of formalin-fixed, paraffin-embedded ESCC primary tumor specimens (normal vs tumor tissues) and 57 paired lymph node metastasis tumor tissues were selected from Linzhou Cancer Hospital (Henan, China). The TMA was constructed as described previously.^[1]^ None of these patients received preoperative treatment. Patient clinical data was included in **Table S2** and **Table S3**. Informed consent was obtained from all patients before the collection of esophageal specimens, and the study was approved by The First Affiliated Hospital of Zhengzhou University Research Ethics Committee (Zhengzhou, China), project number 2023-KY-1084-001.

**Immunohistochemistry (IHC)**

IHC staining was performed by the standard streptavidin–biotin–peroxidase complex method.^[1]^ Briefly, tissue sections with 5μm thickness were cut from the TMA blocks and mounted on microscope slides. The slides were deparaffinized, rehydrated, treated for heat-induced epitope retrieval (HIER) at 95°C for 20 minutes in DAKO citrate target antigen retrieval buffer (pH). and blocked by 10% normal goat serum at room temperature for 30 minutes. Primary antibody used: Polyclonal rabbit-against-Human anti-pan Cytokeratin (pan-CK) antibody (Abcam, Cambridge, UK; dilution 1:400). The slides were then incubated with rabbit polyclonal antibody against DIRAS1 (Abcam) at a dilution of 1:100 at 4°C overnight and subsequently incubated with biotinylated goat anti-rabbit immunoglobulin at a concentration of 1:100 for 30 minutes at 37°C.

**Animal ethics**

Mice of either gender, at age of 4-5 weeks, originally sourced from The Jackson Laboratory, USA (Strain NOD.CB17-Prkdc^scid^/J; NOD SCID) were bred under an AAALAC International accredited program at the Centre for Comparative Medicine Research, HKU under Specific Pathogen Free (SPF) conditions. Research only proceeded following review and approval from the HKU Committee on the Use of Live Animals in Teaching and Research (CULATR# 5753-21) and under licence from the Hong Kong SAR Government’s Department of Health, reference number (20-1288) in DH/HT&A/8/2/3 Pt.17. Animals were housed in individually ventilated cages under a 12:12 dark light cycle within environmentally controlled rooms and were fed *ad libitum* with laboratory diet manufactured by LabDiet, USA.

**Multi-timepoint pulmonary metastasis (mtPM) mouse model**

*In vivo* pulmonary metastatic mice model was established by inoculation of ESCC cells into NOD.CB17-Prkdc^scid^/J (NOD SCID) mice of age 4-5 weeks via intravenous injection. KYSE30-Luc-GFP 1.2×10^6^ cells/100µL PBS for parental mtPM model; fluorescence-activated cell sorting (FACS) sorted KYSE30-Luc-GFP CD44^high^, CD44^low^ or stained control cells 5×10^5^ cells/100µL PBS for CD44-enriched mtPM model; FACS sorted KYSE30-Luc-GFP TACSTD2^high^, TACSTD2^low^ or stained control cells 5×10^5^ cells/100µL PBS for TACSTD2-enriched mtPM model. Bioluminescence signals were detected within 2 hours post-intravenous injection to confirm successful *in vivo* metastasis establishment, in addition to detection at intervals for monitoring and prior to sacrifice followed by tissue collection.

For IHC staining of parental mtPM mice, mice were sacrificed at 2 hours (n=3), 6 hours (n=3), 24 hours (n=4), 48 hours (n=4), 1 week (n=3), 2 months (n=4) and 4 months (n=4). For flow cytometry retrieval of living tumor cells followed by single-cell RNA sequencing, mice were sacrificed at 6 hours (n=3), 48 hours (n=16), 2 months (n=6), and 4 months (n=1). For CD44-enriched mtPM mice, mice were sacrificed at 40 hours (CD44^high^ n=3, CD44^low^ n=5, control n=3) and 2 months (CD44^high^ n=6, CD44^low^ n=6, control n=6). For TACSTD2-enriched mtPM mice, mice were sacrificed at 2 months (TACSTD2^high^ n=4, TACSTD2^low^ n=4, control n=3).

***In vivo* bioluminescence imaging**

Mice were anaesthetized via intraperitoneal injection of 2% Xylazine and 10% Ketamine in PBS (9µL/g of mouse weight), then injection of D-luciferin (GOLDBIO, MO) at 10µL/g of mouse weight and rest for 5 minutes. *In vivo* imaged by IVIS® Spectrum In Vivo Imaging System (Perkin Elmer, MA) supplied with oxygen and constant temperature at 37°C at field D for whole mice for 5 minutes, at field B for freshly dissected tissues for 5 minutes.

**Mouse lung single-cell dissociation and mouse cell depletion**

Mice were sacrificed at designated timepoints post-intravenous injection of tumor cells. Lung tissues were collected and cut into small fragments mechanically and digested into single cells by treatment of liberase for 1 hour at 37°C. Cells were collected by centrifuging at 1,200 rpm for 5 minutes, and treated with 3mL 0.25% Trypsin-EDTA (Gibco, NY) at 37°C for 5 minutes. After neutralizing by 5mL RPMI1640 medium, cells were collected by centrifuging at 1,200rpm for 5 minutes, and then treated with 2mL ACK lysis buffer to remove red blood cells at room temperature for 5 minutes. After centrifugation, cells were resuspended in 980µL full RPMI1640 containing 20µL DNase I at room temperature for 15 minutes. After centrifugation, cells were resuspended in 1mL pre-cold FACS (PBS with 0.5% FBS) buffer, filter cells through 70µm and 40µm cell strainer in order. Perform cell counting by automated hemocytometer. Resuspend cells in pre-cold EDTA-FACS (0.5% EDTA in FACS) buffer ready for flow cytometry population fraction analysis or proceed to remove mouse lung cells for subsequent cell sorting. Tumor cells were recovered by removing mouse lung cells using a magnetic cocktail beads-based Mouse Cell Depletion Kit (Miltenyi Biotec) and LS Columns (Miltenyi Biotec), following the manufacturer’s recommended procedures.

**Antibody staining for flow cytometry**

Collected cells were counted via automated hemocytometer and washed in pre-cold FACS buffer. Antibodies were diluted by pre-cold FACS buffer. Cells were stained in dark with anti-CD44 primary antibody (Cell Signaling Technology, 1:100 dilution) conjugated with Lightning-Link® Alexa Fluor® 647 (Abcam, Cambridge, UK) or isotype control antibody (MBL Life Science, Japan; 1:200 dilution) conjugated with Lightning-Link® Alexa Fluor® 647 (Abcam, Cambridge, UK) at room temperature for 30 minutes with interval flicking every 15 minutes, then washed three times by FACS buffer and centrifugation. Then, resuspend cells in EDTA-FACS buffer and filtered through 40µm filter. For sorting of CD44^high^ and CD44^low^ cells for *in vitro* and *in vivo* assays, cells were stained in dark at room temperature for 30 minutes using anti-CD44 antibody labelled with APC (ThermoFisher, 1:333 dilution) or isotype control antibody (ThermoFisher, 1:333 dilution). After washing and centrifugation, sorted cells were resuspended in pre-cold EDTA-FACS buffer and then passed through 40µm filter. For sorting of TACSTD2^high^ and TACSTD^low^ cells, cells were stained by anti-TACSTD2 primary antibody (AbMart, Shanghai, China, 1:200 dilution) or isotype control antibody (MBL Life Science, Japan, 1:200 dilution) at room temperature for 1 hour with interval flicking every 15 minutes. Washed three times by FACS buffer with centrifugation. Then, stained by secondary antibody with Alexa Fluor Plus 647 (Invitrogen, 1:400 dilution) at room temperature in dark for 20 minutes. Washed three times by pre-cold FACS buffer and centrifugation. Then, sorted cells were resuspend in pre-cold EDTA-FACS buffer and pass through 40µm filter.

**Flow cytometry and cell sorting**

Stained cells or mouse model retrieved cells passed through the BD FACSAria™ SORP Cell Sorter or the BD FACSMelody™ Cell Sorter and sorted based on fluorescence signal intensity. Isotype control tubes were used for gating. Highest 10% and lowest 10% of cells were gated to sort for High and Low groups, respectively. The control group included all cells spanning from lowest to highest gate, passing through flow cytometry sorting machine. Cells were collected in 0.04% FBS-PBS collection medium for subsequent single-cell RNA sequencing; collected in 50% FBA-PBS collection medium supplemented by 3% PS for subsequent *in vitro* functional assays; or collected in 50% FBA-PBS collection medium for subsequent *in vivo* animal experiments. Sorted cells were reloaded immediately to confirm the sorting efficiency.

**Single-cell transcriptome sequencing (scRNA-seq)**

KYSE30-Luc-GFP cells at 5 longitudinal timepoints (i.e. *in vitro* parental stage, *in vivo* 6 hours, 48 hours, 2 months, and 4 months) were collected and retrieved from mtPM mouse model via FACS for single-cell transcriptome sequencing (scRNA-seq). FACS sorted cells were assessed for cell viability and counted prior to sequencing. Multiplex 10× Genomics scRNA-seq was performed using the Chromium Single Cell Gene Expression transcriptome sequencing platform using Chromium™ Single Cell 3’ v2 Library (10× Genomics, USA). A total of 26,712 cells from five timepoints were sequenced.

**scRNA-seq data quality control, dimension reduction, cluster identification and differential gene expression comparison**

All sequencing data passed the quality control checking, performed by Seurat R package (version 3, https://satijalab.org/seurat/)^[2]^ based on number of RNA features and gene counts for single-cell selection and fraction of mitochondria DNA for living cell selection (nFeature_RNA>=200 & nFeature_RNA <=8000 & nCount_RNA >=1000 & percent.mt <=15). A total of 19,986 cells remained for downstream analyses. We then normalized the total counts in each individual cell to 10,000, followed by log transformation to generate the normalized data. ‘Anchor-based’ integration followed by principal component analysis (PCA, 2000 highly variable genes and 50 top PCs) for dimensionality reduction, population clustering and feature selection were performed. Finally, Cell Ranger 6.0.2. Single Cell Analysis Pipelines package was utilized for format transformation at ease for importing into Loupe Cell Browser software for visualization and interactive analyses, uniform manifold approximation and projection (UMAP) plots were used. Differentially expressed gene analyses were calculated by Loupe Cell Browser, defined by *p*-value <0.05. Differentially upregulated genes (*p*-value <0.05, log 2 fold-change >1) of Parental Cluster 1 (Cluster S) were generated for each timepoints (0 hour: 457; 6 hours: 227; 48hr: 631; 2 months: 491; 4 months: 387 genes).

**Selection criteria for metastasis-initiating signatures**

Top 500 differentially upregulated genes in Cluster S were identified and filtered for genes with Log2 count larger than 1 [Log2Cnt>1]. Genes were further filtered to suit the following criteria: “Parental Cluster S [Cnt>0] >60%” AND
“$\frac{Parental Cluster S [Log2Cnt>1]}{Parental [Log2Cnt>1]}>90\%$ “ AND “Parental Non-Cluster S [Log2Cnt>1] <20%” AND “6h [Cnt>0] >80%” AND “Potential surface expressing protein reported in literature and public database”.

**MISs 1+1 filter and efficiency calculation**

10× Loupe cell browser software was utilized for MISs 1+1 filtering. MISs 1+1 filter refers of any 1 from 3 genes from Group A including C19orf33, S100A14, or RHOD (count > 0), plus any 1 from 4 genes from Group B including CST6, CD44, TM4SF1, or TACSTD2 (count > 0). Efficiency of MISs 1+1 filter in defining Cluster S is based on true positive (TP; TruePos_ClusterSPos), false positive (FP; FalsePos_OtherPos), true negative (TN; TrueNeg_OtherNeg), false negative (FN; FalseNeg_ClusterSNeg), positive predictive value (PPV), negative predictive value (NPV), sensitivity, specificity, and accuracy. PPV = TP/(TP+FP). NPV = TN/(TN+FN). Sensitivity = TP/(TP+FN). Specificity = TN/(TN+FP). Accuracy = (TP+TN)/(TP+FP+TN+FN).

**Bulk transcriptome sequencing (RNA-seq)**

Whole transcriptome sequencing was performed on sorted CD44^high^ and CD44^low^ KYSE30 cells. Complementary DNA (cDNA) library was prepared by KAPA mRNA HyperPrep Kit (KR1352-v3.16) targeting purified poly-A containing mRNA, indexed with xGen®Dual Index UMI adapters, and sequenced by Illumina NovaSeq 6000 run for pair-end 151bp sequencing. Sequenced reads were processed by Illumina (bcl2fastq) software and analyzed for bases with quality score ≥Q30 (i.e. 99.9% base call accuracy). Reads were pre-processed and filtered for adapter sequence and high-quality sequence with read length ≥40bp, with <5% unknown bases “N” and <50% bases with quality value ≤11. Then, filtered reads were mapped to the reference genome (i.e. Human Genome GRCh38 downloaded from GENCODE) using STAR (ver. 2.7.8, default parameters) with basic twopassMode. Gene expression was quantified using RSEM (ver. 1.2.31). Differential gene expression analysis was performed using EBSeq (ver. 1.18.0), statistical significance defined as FDR <0.05.

**Gene ontology (GO) and pathway enrichment analyses**

Gene ontology and KEGG pathway enrichment analyses were performed using Sangerbox 3.0,^[3]^ statistical significance defined by *p*-value <0.05.

**Quantitative polymerase chain reaction (qPCR)**

RNA was extracted via standard Trizol extraction method and quantified by BioDrop. Reverse transcription and genomic DNA (gDNA) removal using PrimeScript™ RT Reagent Kit with gDNA Eraser (Perfect Real Time) (TaKaRa, USA; #RR047A), qPCR performed using TB Green® Premix Ex Taq™ (TaKaRa, USA; #RR420A) and detected by LightCycler® 480 System (Roche, Switzerland). Forward and reverse primers were listed in **Table S4**.

**Multiplex IHC (mIHC) staining**

Opal Polaris™ 7-colour multiplex IHC staining (Akoya Bioscience) was performed on FFPE sections (4µm) of PM mouse model lungs at longitudinal timepoints and FFPE sections of ESCC tissue microarray (TMA), i.e. primary tumor (PT) and paired lymph node metastasis (LNM; if present). For TMA with lymph node metastasis, each patient has two paired primary tissues (T1 and T2) and two paired lymph node metastasis tissues (M1 and M2) included. Multiplex staining was performed according to manufacturer’s suggested protocol with optimization of 5 washing steps following each incubation step, and 1:200 dilution for OPAL fluorophore. Each signature corresponded to a labelled fluorophore, i.e. CD44 = OPAL520 (signature A), S100A14 = OPAL570 (signature B), RHOD = OPAL620 (signature C) and TACSTD2 = OPAL690 (signature D). Slides were mounted in dark by Molecular Probes™ ProLong™ Diamond Antifade Mountant (Life Technologies, California, USA) and imaged by PerkinElmer Vectra Polaris™ automated quantitative pathology imaging system (PerkinElmer, Inc., USA).

**Signature expression scoring and differential MISs score (dMISs) formulation**

Multiplex IHC images were analyzed by Phenochart1.1.0. and individual and co-expression scores of signatures were analyzed and computed by inForm2.4.4. software. MISs score included co-expression of CD44 and RHOD (AC) plus co-expression of S100A14 and TACSTD2 (BD), termed as MISs AC_BD. Primary tumor and lymph node metastasis scores correspond to T1_MISs, T2_MISs, M1_MISs, and M2_MISs. Differential MISs score (dMISs) was calculated by average of difference between lymph node MISs score and paired primary tumor MISs score, score capacity scaled from -2 to +2, negative value indicates higher expression in primary tumor, positive value indicates higher expression in lymph node metastasis. Minimum patient dMIS -0.3851; maximum patient dMISs 0.3996; dMISs cutoff points calculated by R survival and survminer packages using command surv_cutpoint, low dMISs defined as ≤-0.08025.

Below showed the equation for dMISs:

$dMISs$ = $\left[ \frac{\left( M1\_MISs -T1\_MISs \right)+\left( M1\_MISs-T2\_MISs \right)+\left( M2\_MISs-T1\_MISs \right)+\left( M2\_MISs-T2\_MISs \right)}{4} \right]$

**Western blotting**

Target proteins were referenced to size ladders (ThermoFisher, MA). Primary antibodies used were as follow: anti-β-catenin (1:1000; Cell Signaling Technology (CST), MA), anti-ZO-1 (1:500; Invitrogen, MA), anti-E-cadherin (1:1000; CST, MA), anti-vimentin (1:1000; CST, MA), anti-slug (1:1000; CST, MA), anti-snail (1:1000; CST, MA) and anti-β-actin (1:5000; Abcam, Cambridge). Secondary antibodies used were as follow: ECL anti-mouse HRP-linked antibody (1:5000; Cytiva, MA), and ECL anti-rabbit HRP-linked antibody (1:5000; Cytiva, MA). Clarity ECL western blotting substrates (Bio-Rad, CA) was used for enhanced chemiluminescence in X-ray film (Carestream Health, NY) exposure.

**Wound-healing assay**

Assess collective cell migration ability in two dimensions. Cells were seeded at confluency with serum-containing complete medium till cell attachment, then starved for 24 hours in serum-free medium prior to wounding incision by a 200µL pipette tip, followed by medium wash and cultured in complete medium for 12 hours. Lesion sites were photographed at 0 hour and 12 hours (CD44-enriched subpopulation) or 24 hours (TACSTD2-enriched subpopulation) under brightfield microscope, analyzed by ImageJ.

**Trans-well migration and invasion assays**

Assess cell migrating and invasive ability. Invasion chamber inserts with pre-coated Matrigel were pre-activated by serum-free medium 3 hours prior to cell seeding. Cells resuspended in 500µL serum-free medium were seeded in the upper chamber insert with 8.0µm pore size polyester (PET) membrane (Falcon), and filled the lower chamber (Falcon) with 750µL medium supplemented with 10% FBS and 1% PS. Cells in migration chamber were incubated for 24 hours in humidified 5% CO₂ incubator. Migrated or invaded cells attached to the lower surface of PET membrane were then washed 3 times with PBS, followed by fixation in 4% paraformaldehyde (PFA) solution for 15 mins at room temperature, and stained in 1% Crystal Violet for 15mins at room temperature. Finally, the chamber insert was washed in tap water and unmigrated or uninvaded cells removed by cotton buds. PET migration or invasion membranes were cut and mounted on glass slides with DPX and photographed under brightfield microscope. Cell numbers were counted using ImageJ software.

**Foci formation**

Access anchorage-dependent growth ability of cells. Sorted signature-enriched cells of 2,000 cells per well were seeded in 6-well plate with full medium and cultured for 8 days. Cells were washed with PBS 3 times, fixed in 4% PFA-PBS 10 minutes at room temperature, then stained in 1% Crystal Violet for 15 minutes at room temperature, finally washed and dried. Wells were photographed, foci were counted and analyzed by ImageJ software.

**Soft agar colony formation assay**

Assess anchorage-independent growth ability of cells. 6-well plate filled with 0.5% low-melting agar-medium mixture as lower layer, 4×10^4^ cells were resuspended in 0.35% low-melting agar-medium mixture as upper layer. Medium was replenished every two days. Cells were cultured for 3 weeks, followed by 0.005% crystal violet dye staining at room temperature for one hour, washed and imaged.

**Spheroid formation assay**

Assess self-renewal ability and multipotency of cells. 1×10^4^ cells were suspended in 1mL of 0.25% methylcellulose-containing DMEM/F12 supplemented with 20ng/mL human recombinant epidermal growth factor (EGF), 10ng/mL human recombinant basic fibroblast growth factor (bFGF), 4µg/mL insulin, 2% v/v B27 and 1% v/v PS. Cells were seeded in ultra-low attachment surface 6-well plate (Corning), medium was added every two days and cultured for 3 weeks. Total number of spheroids formed per well was counted, then analyzed. Spheroids were imaged under microscope at total 100× magnification.

**Reactive oxygen species (ROS) assay**

Assess oxidative stress level in cell using the Reactive Oxygen Species Assay Kit (Beyotime, China). Collected cells were washed twice followed by incubation in H₂O₂ (166ng/mL)-containing serum-free medium for 30 minutes at humidified 37°C incubator. Then, washed twice in serum-free medium, incubated in 10µM DCFH-DA containing serum-free medium per 10 million cells/mL at humidified 37°C incubator in dark for 20 minutes, tilted to mix every 5 minutes. Washed in serum-free medium three times then resuspended in FACS buffer. Flow cytometry analysis for DCF positive level in cell.

**Apoptosis assay**

Assess apoptosis rate of cells. Cells collected from FACS were seeded for plate attachment with full RPMI1640 medium, then washed with PBS to remove unattached dead cells. Different concentrations of hydrogen peroxide (H₂O₂) were added to seeded cells for 10 hours to stimulate reactive oxygen species production. Apoptosis detection kit (BD Biosciences, NJ) was used. Cells were trypsinised and collected. Annexin V labelled with PE fluorescence and 7AAD were added in dark for 15 minutes according to manufacturer’s instruction. Cells were analyzed by flow cytometry for early and late apoptosis stage. Comparisons between groups were performed by statistical analyses.

**Statistical analyses**

IBM® SPSS® Statistics 27 (IBM Corporation, Armonk, NY) was utilized for statistical analyses. Shapiro-Wilk test was performed to verify normality of data distribution, for non-normally distributed data, two-tailed non-parametric Kruskal-Wallis H test (ranked one-way ANOVA) and Mann-Whitney U test were performed to demonstrate statistical differences between different groups. Spearman’s Rho rank correlation test was performed to demonstrate correlation of two parameters. Cox regression survival curve was performed to classify parameters into two groups and plot the survival curve. Statistical significance defined by *p* <0.05, indicated by ****: *p*<0.0001, ***: *p*<0.001, **: *p*<0.01, *: *p*<0.05, and ns: *p*>0.05.

**Data visualization**

ScRNA-seq cell spatial distributions were displayed at UMAP plots, generated by R package and 10X Loupe Cell Browser software. Statistical analyses results were visualized by GraphPad Prism 9 (GraphPad Software, San Diego, USA).

**Graphical illustration**

PowerPoint and Sketchbook software were utilized for creation of graphics for illustrations.

**Materials availability**

Customized antibodies generated in this study have been deposited to AbMart, #36813-1-1M1/2L8.

**Data availability**

Raw and processed bulk RNA-seq and scRNA-seq data were deposited to the NCBI GEO data repository with accession numbers GSE249058 (<https://www.ncbi.nlm.nih.gov/geo/query/acc.cgi?acc=GSE249058>) for this study; GSE249056, GSM7925717, and GSM7925718 for bulk RNA-seq; GSE249057, GSM7925719, GSM7925720, GSM7925721, GSM7925722, and GSM7925723 for scRNA-seq.

**References**

1 Ming, X.-Y. *et al.* Integrin α7 is a functional cancer stem cell surface marker in oesophageal squamous cell carcinoma. *Nature communications* **7**, 13568 (2016).

2 Stuart, T. *et al.* Comprehensive integration of single-cell data. *cell* **177**, 1888-1902. e1821 (2019).

3 Shen, W. *et al.* Sangerbox: a comprehensive, interaction‐friendly clinical bioinformatics analysis platform. *Imeta* **1**, e36 (2022).
